# Proteasome subunit α1 overexpression preferentially drives canonical proteasome biogenesis and enhances stress tolerance in yeast

**DOI:** 10.1038/s41598-019-48889-5

**Published:** 2019-08-27

**Authors:** Lauren A. Howell, Anna K. Peterson, Robert J. Tomko

**Affiliations:** Department of Biomedical Sciences, Florida State University College of Medicine, Tallahassee, Florida 32306 USA

**Keywords:** Proteasome, Proteasome, Proteasome

## Abstract

The 26S proteasome conducts the majority of regulated protein catabolism in eukaryotes. At the heart of the proteasome is the barrel-shaped 20S core particle (CP), which contains two β-rings sandwiched between two α-rings. Whereas canonical CPs contain α-rings with seven subunits arranged α1-α7, a non-canonical CP in which a second copy of the α4 subunit replaces the α3 subunit occurs in both yeast and humans. The mechanisms that control canonical *versus* non-canonical CP biogenesis remain poorly understood. Here, we have repurposed a split-protein reporter to identify genes that can enhance canonical proteasome assembly in mutant yeast producing non-canonical α4-α4 CPs. We identified the proteasome subunit α1 as an enhancer of α3 incorporation, and find that elevating α1 protein levels preferentially drives canonical CP assembly under conditions that normally favor α4-α4 CP formation. Further, we demonstrate that α1 is stoichiometrically limiting for α-ring assembly, and that enhancing α1 levels is sufficient to increase proteasome abundance and enhance stress tolerance in yeast. Together, our data indicate that the abundance of α1 exerts multiple impacts on proteasome assembly and composition, and we propose that the limited α1 levels observed in yeast may prime cells for alternative proteasome assembly following environmental stimuli.

## Introduction

**Figure 1:**
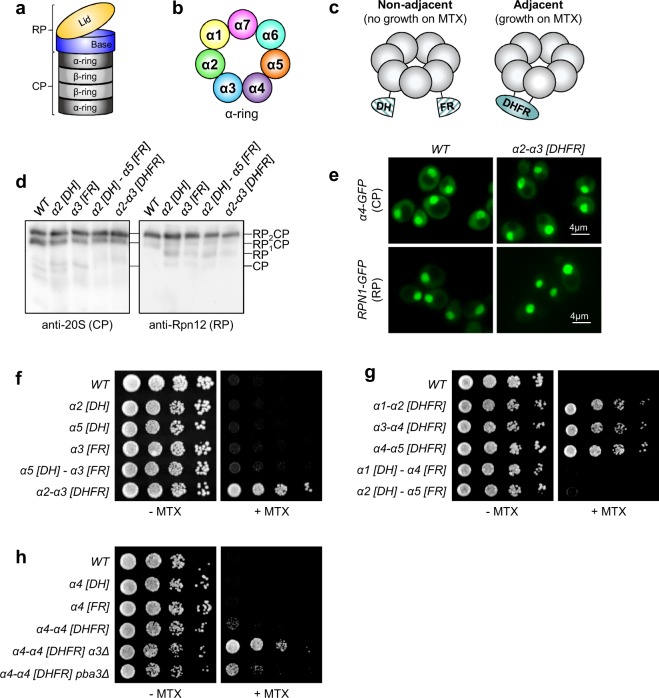
Split-DHFR complementation reports on canonical and non-canonical CP subunit arrangements. (**a**) Illustration of the 26S proteasome depicting the major subcomplexes. RP, regulatory particle; CP, core particle. (**b**) Illustration of the canonical arrangement of α-subunits within the α-ring of the 20S core particle. (**c**) Schematic depicting split-DHFR complementation to monitor proteasome subunit juxtaposition *in vivo*. (**d**) Cell extracts from yeast strains expressing α-subunits fused with N- or C-terminal DHFR fragments (designated [DH] or [FR], respectively) from their chromosomal loci were separated by non-denaturing PAGE and immunoblotted with antibodies against the 20S CP (left) and the RP lid subunit Rpn12 (right). The positions of doubly-capped CP (RP_2_CP), singly-capped CP (RP_1_CP), RP, and CP are shown. Full-length blots are presented in Supplementary Fig. S8. (**e**) Subcellular localization of the CP subunit α4-GFP (top row) or RP subunit Rpn1-GFP (bottom row) is unaffected by expression of the *α2-α3 [DHFR]* reporter pair. (**f–h**) Equal numbers of cells from the indicated yeast strains were spotted in six-fold serial dilutions on synthetic complete plates lacking or containing methotrexate (MTX) and incubated for three days at 30 °C.

The ubiquitin-proteasome system (UPS) is the primary mechanism for regulated protein catabolism in eukaryotes^1,2^. UPS substrates are typically first modified with a chain of the small protein ubiquitin, which targets substrates for degradation by the 26S proteasome. The 26S proteasome is a large, multisubunit ATP-dependent peptidase complex composed of a barrel-shaped 20S core particle (CP) that can be capped on one or both ends by the 19S regulatory particle (RP) (Fig. 1a). The RP mediates the deubiquitination and unfolding of substrates, and translocates them into the CP. The CP then cleaves substrates into short peptides. The canonical CP complex consists of four axially stacked heteroheptameric rings: two inner β-rings sandwiched between two outer α-rings. In the canonical CP, the α-ring is composed of seven α-subunits (α1-α7, Fig. 1b), whereas the β-ring is comprised of seven β-subunits (β1-β7). The β-rings house the peptidase activities, which are encoded by the β1, β2, and β5 subunits.

In addition to the canonical CP, several alternative CP species with distinct subunit compositions can be formed via substitution of canonical CP subunits with alternative isoforms. In mammals, four alternative β-subunits have been identified: β1i, β2i, β5i, and β5t. These paralogs can substitute for their respective canonical β-subunits within the CP to form two distinct proteasome isoforms known as the immunoproteasome^3,4^ and thymoproteasome^5,6^. The immunoproteasome contains β1i, β2i, and β5i in place of β1, β2, and β5, and is constitutively expressed in immune cells. Assembly of the immunoproteasome is induced in other cell types upon stimulation with interferon γ. The thymoproteasome contains β1i, β2i, and β5t, and is expressed exclusively in cortical thymic epithelial cells. The immunoproteasome and thymoproteasome display altered proteolytic activities with respect to canonical CPs. These altered activities enhance peptide generation for antigen presentation by major histocompatibility complex class I molecules and for positive selection of CD8 + T cells, respectively^7^.

An alternative α-subunit, α4s, has been identified in the testes of many organisms and is most abundant in spermatids and mature sperm^8–10^. Substitution of this paralog for the canonical α4 subunit results in the formation of the spermatoproteasome. Although the exact role of the spermatoproteasome remains unclear, it is essential for fertility^11^ and is thought to mediate the degradation of histones and other sperm-specific substrates that are essential for efficient spermatogenesis^10^.

In the budding yeast *Saccharomyces cerevisiae*, all CP genes are essential for viability with the exception of the gene encoding the α3 subunit^12^. Yeast lacking α3 synthesize a non-canonical CP, referred to as the α4-α4 CP, in which a second copy of the canonical α4 subunit is incorporated into the position normally occupied by the absent α3 subunit^13^. Such α4-α4 CPs also occur in human cells^14^. Notably, overexpression of the oncogenic kinases ABL or ARG is associated with increased abundance of α4-α4 CPs, whereas the tumor suppressor BRCA1 negatively regulates α4-α4 CP formation^14^. These observations suggest that α4-α4 CPs play a role in carcinogenesis or maintenance of the tumor phenotype. Indeed, alterations in α3 and α4 levels suggestive of α4-α4 CP formation (*i*.*e*. reduced α3, increased α4) have been identified in some cancer types^14^, and the assembly of α4-α4 CPs confers resistance to heavy metal stress in both yeast and humans^14,15^.

In yeast and humans, the abundance of α4-α4 CPs is primarily controlled by a pair of evolutionarily conserved proteasome assembly chaperones, Pba3 and Pba4 (PAC3 and PAC4 in humans)^14–19^. These chaperones form a heterodimer (Pba3-4) that promotes α3 incorporation over a second copy of α4 to yield canonical CPs. Under conditions commonly associated with the tumor phenotype, such as increased proteasome biogenesis^20–22^ and oxidative stress^23^, Pba3-4 becomes limiting for proteasome assembly, resulting in enhanced formation of α4-α4 CPs^14^. In yeast and humans, deletion of *PBA3* or *PBA4* results in formation of both canonical and non-canonical CPs. However, it is unknown what governs whether α3 or α4 incorporates into the α3 position during such limiting chaperone activity.

Despite the emerging importance of the non-canonical α4-α4 CP, a comprehensive analysis of genes influencing assembly of canonical *versus* non-canonical CPs has not yet been performed. This is due in part to a lack of suitable methods to discriminate the *in vivo* subunit composition of the proteasome in a high-throughput format. Thus far, experimental detection of α4-α4 CP assembly has relied on biochemical analyses of purified proteasomes or engineered disulfide crosslinking of α-subunits^13–15^ in cell extracts visualized by SDS-PAGE, neither of which are amenable to high-throughput analyses or genetic screening.

In this study, we have repurposed a split-dihydrofolate reductase (DHFR) reporter to perform a genome-wide screen for genes that enhance canonical CP assembly in *pba3Δ* yeast. We identified the proteasome subunit α1 as a preferential enhancer of canonical CP assembly and demonstrate that the relative abundance of α1 governs the ratio of canonical to non-canonical CPs when Pba3-4 activity is limiting. Further investigation revealed that the abundance of α1 regulates the steady-state level of 26S proteasomes and stress tolerance in yeast. Integration of these findings with previous studies suggests that incorporation of α1 into the assembling α-ring prior to a second copy of α4 confers commitment to canonical CP biogenesis.

## Results

### Split-DHFR complementation can report on canonical and non-canonical proteasome subunit arrangements *in vivo*

To date, no systematic *in vivo* analysis of genes influencing CP composition has been conducted. Toward this goal, we repurposed a protein complementation assay based on the reconstitution of split-DHFR^24–26^ to establish a growth-based reporter to monitor the juxtaposition of particular pairings of proteasome subunits (exemplified in Fig. 1c). This survival-selection assay employs a mutant split-DHFR reporter harboring mutations that confer resistance to the DHFR inhibitor methotrexate (MTX). MTX potently inhibits the endogenous yeast dihydrofolate reductase, Dfr1, leading to growth inhibition. The N- and C-terminal fragments of the mutant split-DHFR reporter (hereafter referred to as [DH] and [FR], respectively) are fused to two query proteins. An interaction between the two query proteins reconstitutes the mutant DHFR and confers growth on media containing MTX. We hypothesized that fusion of these DHFR fragments to pairs of α-subunits would report on their juxtaposition primarily within fully assembled CPs, which greatly outnumber proteasomal assembly intermediates in rapidly growing cells^13,15,17^.

The C-terminus of each α-subunit contains a conserved α-helix that is solvent exposed and points outward from the CP (Supplementary Fig. S1a,b). These C-terminal α-helices are clearly resolved in both atomic and cryo-electron microscopy structures, indicating they are largely immobile. We engineered yeast strains expressing α-subunits with C-terminal [DH] or [FR] fusions from their native chromosomal loci. The [DH] and [FR] fragments were connected to their cognate α-subunits via a flexible linker sequence. The length of this linker provides sufficient reach to allow DHFR reconstitution between adjacent α-subunits within the CP (~42–62 Å between α-subunit C-terminal carboxylate carbons, Supplementary Fig. S1a). However, the linker is not long enough to permit complementation between non-adjacent α-subunits (~86–100 Å between C-terminal carboxylate carbons, Supplementary Fig. S1a) or subunits in different α-rings (~129–158 Å between C-terminal carboxylate carbons, Supplementary Fig. S1b).

We first confirmed that the α-subunit DHFR fusion proteins were well tolerated. Native PAGE analysis of extracts from yeast harboring the *α2 [DH]*, *α3 [FR]*, or *α5 [FR]* alleles either alone or in pairs (*α2-α3 [DHFR]*, *α2 [DH] - α5 [FR]*) revealed no obvious changes in the abundance of doubly (RP_2_CP) or singly (RP_1_CP) capped proteasomes compared to wild-type (WT) cells (Fig. 1d and Supplementary Fig. S1c), suggesting they do not impair proteasome assembly. We next tested whether split-DHFR alleles impacted proteasome subcellular localization. We introduced the *α2-α3 [DHFR]* pair into yeast strains expressing GFP fusions to the CP subunit α4^27^ or the RP base subunit Rpn1 from their respective chromosomal loci, and visualized proteasome localization by fluorescence microscopy. No gross changes in subcellular localization of α4-GFP or Rpn1-GFP were observed when the *α2-α3 [DHFR]* subunit pair was present (Fig. 1e). Together, these observations suggest that α-subunit DHFR fusions are well tolerated.

We next examined the growth of these yeast strains in the presence and absence of MTX. No growth defects were evident in yeast expressing any of the DHFR fusions at 30 °C on complete media lacking MTX (Fig. 1f, left panel). In the presence of MTX, the *α2-α3 [DHFR]* yeast grew readily (Fig. 1f, right panel). Similar results were obtained when the [DH] and [FR] fragments were swapped between the respective α-subunits (Supplementary Fig. S1d). In contrast, no growth was observed for WT cells or cells expressing the *α2 [DH]*, *α5 [DH]*, or *α3 [FR]* fusions, indicating complementation was dependent on the presence of both fragments. Importantly, cells expressing the *α5 [DH] - α3 [FR]* pair also failed to grow, despite the *α5 [DH]* and *α3 [FR]* fusions conferring growth on MTX when fused to adjacent subunit pairs (Supplementary Fig. S1e and Fig. 1g, respectively). We observed similar results with other adjacent and non-adjacent α-subunit pairs (Fig. 1g). Together, these data confirm that only directly adjacent α-subunit DHFR fusions reconstitute DHFR activity, consistent with our modeling.

We next sought to determine if this approach could be used to report on changes to the canonical α-subunit arrangement. In yeast, deletion of the *α3* gene or the gene encoding the proteasomal assembly chaperone *PBA3* promotes formation of α4-α4 CPs at 100% and ~50% frequency, respectively^13,15^. We generated an *α4-α4 [DHFR]* reporter strain by introducing centromeric plasmids encoding *α4 [DH]* and *α4 [FR]* into an *α4Δ* strain by plasmid shuffle^28^. As anticipated, the *α4-α4 [DHFR]* reporter strain harboring WT copies of *α3* and *PBA3* showed minimal growth on media containing MTX (Fig. 1h). In contrast, the reporter strain grew readily when either *α3 or PBA3* were deleted. Importantly, *α3Δ* cells grew better than *pba3Δ* cells, in agreement with the known abundances of α4-α4 CPs in these mutants. Taken together, these data demonstrate that split-DHFR complementation can report on the relative abundances of both canonical and non-canonical CP subunit arrangements *in vivo*.

### A genome-wide screen in pba3Δ yeast identifies the proteasome subunit α1 as an enhancer of α2-α3 juxtaposition

**Figure 2:**
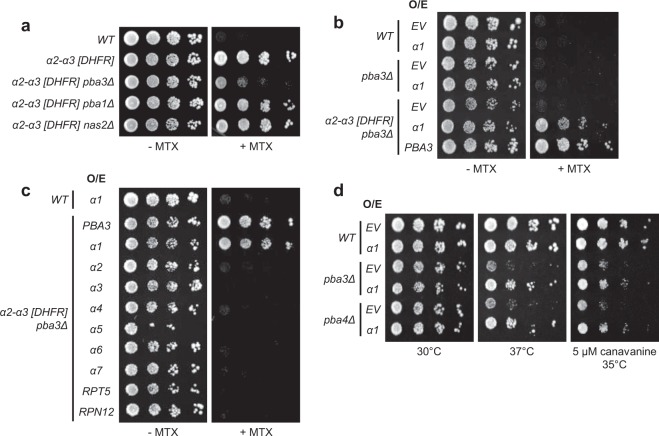
A genome-wide screen identifies proteasome subunit α1 as an enhancer of α2-α3 juxtaposition in *pba3Δ* cells. (**a**) Equal numbers of cells from the indicated yeast strains were spotted in six-fold serial dilutions on synthetic complete plates lacking or containing MTX and incubated for three days at 30 °C. (**b**) Equal numbers of WT, *pba3∆*, or *α2-α3 [DHFR] pba3∆* cells were transformed with empty vector (EV) or with high-copy plasmids encoding *α1* or *PBA3*. Transformants were spotted in six-fold serial dilutions onto synthetic complete plates lacking or containing MTX and incubated for three days at 30 °C. (**c**) Equal numbers of WT or *α2-α3 [DHFR] pba3∆* cells expressing the indicated proteins from high-copy plasmids were spotted in six-fold serial dilutions onto synthetic complete plates lacking or containing MTX and incubated for three days at 30 °C. (**d**) Equal numbers of cells from the indicated yeast strains expressing empty vector (EV) or α1 from a high-copy plasmid were spotted in six-fold serial dilutions on the indicated media and incubated as shown for three days.

We utilized *pba3Δ* cells as a model of chaperone limitation in an effort to identify novel regulators of CP subunit composition. Because these cells form ~50% canonical and ~50% non-canonical CPs, it should be possible to search for genes that bias toward either canonical or non-canonical CP assembly. We initially attempted to use the *α4-α4 [DHFR]* reporter strain to search for genes that promoted enhanced formation of α4-α4 CPs. However, leaky growth of this reporter strain on MTX (Fig. 1h) hampered this approach. We instead decided to query for genes that enhanced canonical CP assembly in *pba3Δ* cells using the *α2-α3 [DHFR]* reporter pair. As expected, deletion of *PBA3* resulted in poor growth on media containing MTX due to reduced incorporation of α3 into the CP. This effect was specific for *PBA3*, as deletion of genes encoding the unrelated CP assembly chaperone *PBA1* or the RP assembly chaperone *NAS2* had no effect on growth on MTX (Fig. 2a).

We next introduced the yeast genomic tiling library^29^ at ~12-fold depth of coverage into the *α2-α3 [DHFR] pba3Δ* reporter strain. This high-copy library consists of an ordered array of plasmids containing yeast genomic DNA fragments that together cover ~97% of the *S*. *cerevisiae* genome. We then plated transformants on media containing MTX to identify clones whose genomic fragments promoted enhanced juxtaposition of α2 and α3, as gauged by an increase in growth on MTX. Surprisingly, only a single plasmid was found to confer enhanced growth on MTX, with the exception of the positive control plasmid encoding *PBA3*. Sequencing and subcloning of individual genes encoded by the recovered plasmid identified the proteasome subunit *α1* as the gene enhancing canonical incorporation of α3 adjacent to α2 (Fig. 2b and Supplementary Fig. S2a). Overexpression of any of the remaining six α-subunits, the RP base subunit Rpt5, or the RP lid subunit Rpn12 failed to confer any appreciable growth on MTX (Fig. 2c), indicating the effect was highly specific to α1. We confirmed via immunoblotting that these subunits were overproduced (we did not verify overexpression of α5, as it is not recognized by the 20S antibody used), indicating that the lack of MTX growth observed in Fig. 2c was not due to failed expression (Supplementary Fig. S2b–d). Although α3 overexpression would be expected to enhance α3 incorporation by mass action, it would compete for insertion with the *α3 [FR]* fusion, explaining why it was not identified in our screen. Similarly, overexpression of α2 would complete for insertion with the *α2 [DH]* fusion and thus would obscure any potential effects of α2 overproduction.

Deletion of the genes encoding Pba3 or its heterodimeric binding partner Pba4 confers sensitivity to elevated temperatures and the amino acid analog *L*-canavanine due to the resultant imbalance of canonical and non-canonical CP biogenesis^15^. Interestingly, we found that overproduction of α1 in *pba3Δ* and *pba4Δ* yeast partially rescued the temperature and *L*-canavanine sensitivity in both mutants (Fig. 2d). This effect was not observed upon overproduction of a different α-subunit, α7 (Supplementary Fig. S2e). Together with the data from our screen, these findings are consistent with the possibility that α1 overproduction enhances canonical CP levels when chaperone activity is limiting.

### The stoichiometry of α1 governs the ratio of canonical to non-canonical CPs in pba3Δ cells

**Figure 3:**
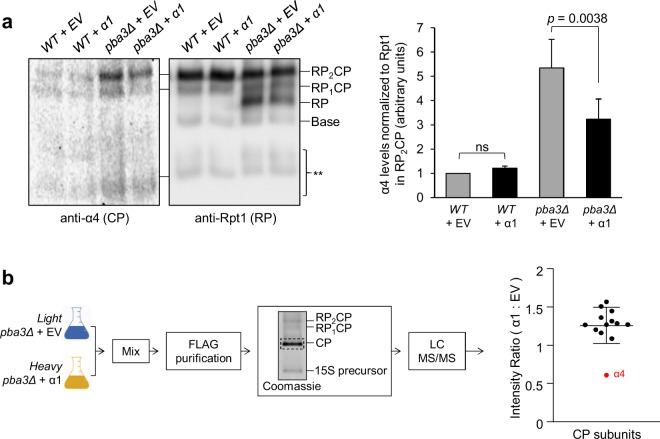
Overexpression of α1 alters the composition of the CP in *pba3Δ* cells. (**a**) Cell extracts of WT or *pba3Δ* yeast expressing empty vector (EV) or α1 from a high-copy plasmid were separated by non-denaturing PAGE and immunoblotted with antibodies against the α4 subunit (left) and the RP base subunit Rpt1 (right). The positions of doubly-capped CP (RP_2_CP), singly-capped CP (RP_1_CP), RP, and the RP base are shown. Shown to the right is quantification of α4 levels in fully assembled proteasomes (RP_2_CP) normalized to the corresponding RP_2_CP band in the Rpt1 blot (*n* = 8; error bars = s.e.m.; ns = not significant, *p* = 0.9932). **, CP assembly intermediates. Full-length blot is presented in Supplementary Fig. S8. (**b**) Workflow of SILAC analysis with resulting peptide ion intensity ratios of CP subunits. Cells lacking *PBA3* and harboring the indicated high-copy plasmids were metabolically labeled as described in Materials and Methods and lysed. Equal amounts of protein were mixed before immunoaffinity purification of the CP for LC-MS/MS analysis. The average peptide ion intensity ratios (α1: empty vector) for each CP subunit are shown, with the α4 subunit highlighted in red. Error bars indicate the s.d. of the intensity ratios of the CP subunits. EV, empty vector. Full-length gel is presented in Supplementary Fig. S8.

We next sought to directly examine the effect of α1 overproduction on the assembly of canonical *versus* non-canonical CPs. We first tested whether α1 overproduction enhances formation of canonical CPs in *pba3Δ* cells, as suggested by our screen. If this is the case, 26S proteasomes from *pba3Δ* cells would be expected to contain less α4 on average when α1 was overproduced. In native PAGE-separated extracts from WT cells, no difference in the abundance of α4 was observed when α1 was overproduced (Fig. 3a). Consistent with previous findings^14,15^, the abundance of α4 was increased substantially in extracts of *pba3Δ* cells expressing empty vector, compared to WT expressing empty vector. Importantly, upon overproduction of α1, the abundance of α4 was significantly reduced, suggesting that α1 overproduction enhances the formation of canonical CPs compared to non-canonical CPs.

In agreement with our observations in Fig. 3a, we observed a similar decrease in α4 levels in CPs purified from cells overproducing α1 using stable isotope labeling with amino acids in cell culture (SILAC)-coupled mass spectrometry. We first introduced empty vector or a high-copy plasmid encoding α1 into *pba3Δ* yeast expressing β5-3xFLAG from the chromosomal locus as a purification handle. This strain did not exhibit any overt growth defects under standard growth conditions (Supplementary Fig. S3). Transformants were then cultured in media containing either light or heavy lysine to label cellular proteins. Extracts of each strain were then prepared and mixed together in equal amounts. We next purified the CP from the combined extracts via FLAG affinity, eluted the CP with excess 3xFLAG peptide, and separated the purified product via native PAGE. We excised the band corresponding to the CP, which was subsequently digested with trypsin and analyzed via liquid chromatography-tandem mass spectrometry (LC-MS/MS). Changes in CP subunit composition were thereby evident as a deviation in the relative heavy-to-light peptide ion intensity ratio from the average ratio for all subunits. We observed a small increase in abundance of all CP subunits upon α1 overexpression compared to empty vector, with the sole exception of the α4 subunit (Fig. 3b). The abundance of α4 present in CPs from α1-overproducing cells was approximately 0.6 that of empty vector, indicating that α4 was approximately two-fold less abundant in CPs from α1-overproducing cells.

**Figure 4:**
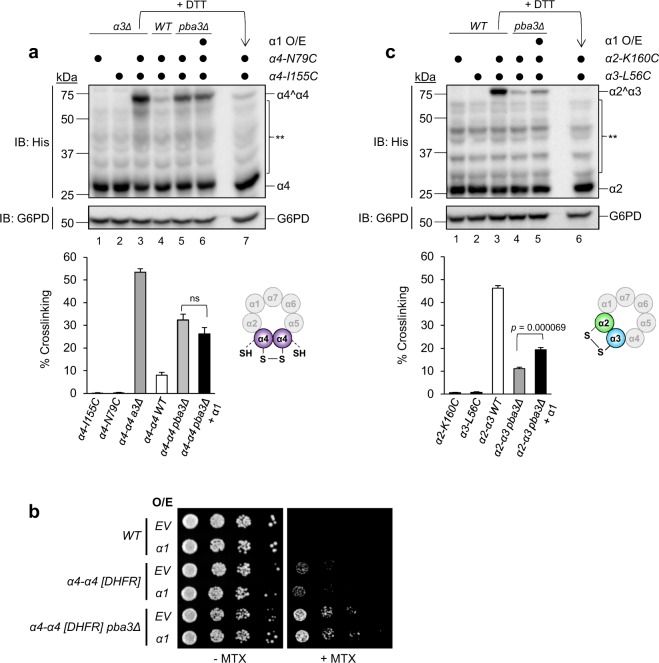
The stoichiometry of α1 governs the ratio of canonical to non-canonical proteasomes in *pba3Δ* cells. (**a**) Extracts of WT and *pba3Δ* yeast overexpressing α1 or not and harboring the indicated cysteine substitutions were crosslinked as described in Materials and Methods, followed by separation by non-reducing SDS-PAGE and immunoblotting with antibodies against His (α4) and G6PD (loading control). Crosslinks between adjacent α4 subunits are indicated as α4^α4 and are quantified below (*n* = 6; error bars = s.e.m.; ns = not significant, *p* = 0.1754). **Nonspecific cross-reactive bands. Full-length blot is presented in Supplementary Fig. S8. (**b**) Equal numbers of cells from the indicated yeast strains expressing empty vector (EV) or α1 from a high-copy plasmid were spotted in six-fold serial dilutions on synthetic complete plates lacking or containing MTX and incubated for three days at 30 °C. (**c**) Extracts of WT and *pba3Δ* yeast overexpressing α1 or not and harboring the indicated cysteine substitutions were crosslinked as described in Materials and Methods, followed by separation by non-reducing SDS-PAGE and immunoblotting with antibodies against His (α2) and G6PD (loading control). WT cells served as a positive control for crosslinking (lane 3), which was evident as a high-molecular weight species that could be near-fully ablated by treatment of the crosslinked extract with DTT prior to electrophoresis (lane 6).Crosslinks between adjacent α2 and α3 are indicated as α2^α3 and are quantified below (*n* = 6; error bars = s.e.m.). **Nonspecific cross-reactive bands. Full-length blot is presented in Supplementary Fig. S8.

We next utilized engineered disulfide crosslinking to test whether the decrease in α4 was due to reduced incorporation of a second copy of α4. Copper-induced disulfide crosslinking between two engineered cysteines introduced at the α4-α4 or α2-α3 interface has been used successfully in the past to demonstrate the formation of canonical and non-canonical CPs, respectively^13–15^. Following this approach, we introduced N79C and I155C substitutions alone or together into the coding sequence of α4 in WT or *pba3Δ* yeast to allow for relative quantification of α4-α4 juxtaposition by crosslinking, which was evident as a DTT-sensitive high molecular weight species upon non-reducing SDS-PAGE (Fig. 4a). As observed previously^13,15^, crosslinking was dependent upon the presence of both engineered cysteines (Fig. 4a, lanes 1, 2, and 3). Consistent with previous reports^14,15^, analysis of non-reduced extracts of *pba3Δ* cells expressing empty vector demonstrated a modest increase in crosslinked α4 upon oxidation with Cu^2+^ compared to WT expressing empty vector (Fig. 4a, lane 5 vs. lane 4). To our surprise, however, only a minimal reduction in α4-α4 crosslinking was observed upon overproduction of α1 in *pba3Δ* cells (Fig. 4a, lane 6 vs. lane 5). Consistent with this, the growth of the *α4-α4 [DHFR] pba3Δ* reporter strain on MTX was unaffected by α1 overproduction (Fig. 4b). Although these observations do not completely rule out the possibility that α1 overexpression suppresses α4-α4 proteasome assembly, they suggest that dilution of α4-α4 proteasomes resulting from elevated canonical CP biogenesis is the more dominant mechanism.

We tested this hypothesis directly using a similar approach in which α2-K160C and α3-L56C substitutions permit disulfide crosslinking only when α2 and α3 are directly juxtaposed (Fig. 4c, lanes 1–3). In agreement with our earlier data (Fig. 2a), analysis of non-reduced extracts of *pba3Δ* cells expressing empty vector demonstrated a decrease in crosslinking between α2 and α3 upon oxidation with Cu^2+^ compared with WT cells expressing empty vector (Fig. 4c, lane 4 vs. lane 3). Upon overexpression of α1 in *pba3Δ* cells, we observed a 1.76-fold increase in crosslinking compared to *pba3Δ* cells expressing empty vector (Fig. 4c, lane 5 vs. lane 4). This was consistent with the results of our initial screen (Fig. 2b) and the reduction in α4 observed via native PAGE-immunoblotting and SILAC-coupled mass spectrometry (Fig. 3). Together, these data indicate that α1 overproduction preferentially enhances canonical CP biogenesis in *pba3Δ* cells.

**Figure 5:**
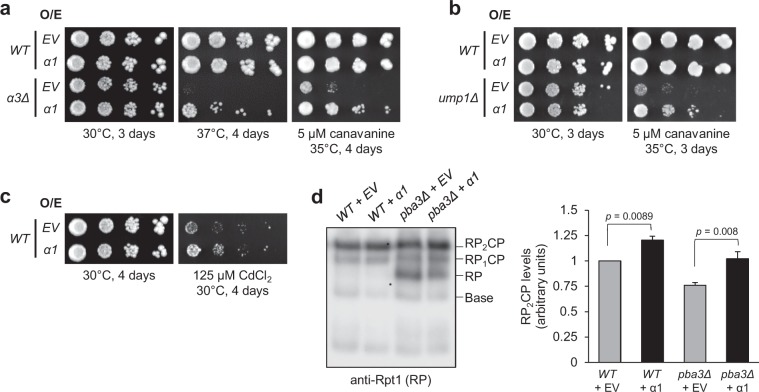
Overexpression of α1 enhances resistance of WT and CP mutant yeast to proteotoxic stress. (**a**–**c**) Equal numbers of cells from the indicated yeast strains expressing empty vector (EV) or α1 from a high-copy plasmid were spotted in six-fold serial dilutions on the indicated media and incubated as specified. (**d**) Cell extracts of WT or *pba3Δ* yeast expressing empty vector (EV) or α1 from a high-copy plasmid were separated by non-denaturing PAGE before immunoblotting with antibodies against the RP base subunit Rpt1. The positions of doubly-capped CP (RP_2_CP), singly-capped CP (RP_1_CP), RP, and base subcomplex are shown. Quantification of Rpt1 levels in fully assembled proteasomes (RP_2_CP) is shown to the right (*n* = 10; error bars = s.e.m.). Full-length blot is presented in Supplementary Fig. S8.

We hypothesized that if α1 overproduction solely and specifically drives canonical CP formation, then it should have no effect in *α3Δ* cells, which cannot assemble canonical CPs due to the absence of α3. However, we unexpectedly found that overexpression of α1 suppressed both the temperature and *L*-canavanine sensitivity of *α3Δ* cells^15^ (Fig. 5a). Similarly, α1 overexpression suppressed the temperature and *L*-canavanine sensitivity of cells lacking the CP assembly chaperone Ump1^30^ (Fig. 5b), and enhanced growth of WT cells on media containing the heavy metal salt CdCl_2_ (Fig. 5c). This suggested that overproduction of α1 had a more general impact on proteasome levels. Indeed, when α1 was overexpressed, we observed a loss of free RP and a corresponding increase in 26S proteasomes (~20% and ~34% in WT and *pba3Δ* cells, respectively), consistent with enhanced CP assembly (Fig. 5d and Supplementary Fig. S4). Taken together with our compositional analyses (Figs 3 and 4), these data suggest that α1 overproduction acts as a general enhancer of CP biogenesis, but it preferentially promotes canonical CP biogenesis when either canonical or non-canonical CPs can be assembled.

### The α1 subunit is stoichiometrically limiting for α-ring assembly in yeast

**Figure 6:**
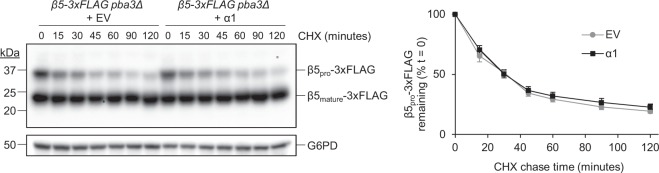
The rate of proteasome core particle maturation is unaffected by α1 overexpression. Yeast expressing β5-3xFLAG from the chromosomal locus in a *pba3Δ* background were transformed with empty vector (EV) or a high-copy plasmid encoding α1. Log phase cells were treated with 250 μg/mL cycloheximide (CHX) to block new protein synthesis and harvested at the indicated times. Cell extracts were separated by SDS-PAGE before immunoblotting with antibodies against FLAG and G6PD (loading control). Bands corresponding to the precursor species (β5_pro_-3xFLAG) were normalized to the G6PD loading control. Quantification of the remaining propeptide as a percentage of propeptide present at time = 0 is shown to the right (*n* = 8; error bars = s.e.m.). Full-length blot is presented in Supplementary Fig. S8.

We next sought to identify the mechanism(s) by which α1 overexpression could enhance CP assembly. Recently, it was shown in human cells that incorporation of the α2 subunit was completely dependent upon the prior incorporation of α1^31^. We thus considered that α1 incorporation could be rate-limiting for CP assembly. Upon completion of CP assembly, propeptides present on several CP β-subunits are removed^32^. This processing event is evident as a bandshift by SDS-PAGE, providing a convenient reporter for completion of CP assembly. We used a cycloheximide chase assay to monitor the rate of propeptide cleavage from β5-3xFLAG (Supplementary Fig. S3) in *pba3Δ* cells expressing empty vector or overproducing α1 (Fig. 6). Consistent with our observations that α1 overexpression increases proteasome abundance, we saw a small but reproducible increase in the levels of mature, fully processed β5 (Supplementary Fig. S5). However, no difference in the rate of propeptide cleavage was observed in cells overproducing α1 (Fig. 6). Thus, α1 incorporation is not a rate-limiting step in CP biogenesis.

**Figure 7:**
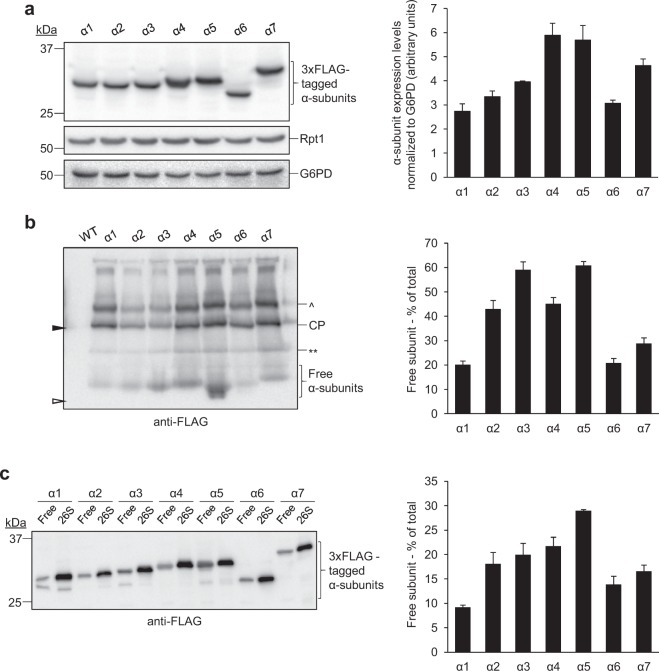
Proteasome subunit α1 is stoichiometrically limiting for proteasome α-ring assembly. (**a**) Whole cell lysates of yeast strains expressing the indicated 3xFLAG-tagged α-subunits from their chromosomal loci were separated by SDS-PAGE and immunoblotted with antibodies against FLAG, Rpt1, or G6PD (loading control). Quantification of α-subunit expression levels, normalized to the G6PD loading control, is shown to the right (*n* = 3; error bars = s.e.m.). Full-length blot is presented in Supplementary Fig. S8. (**b**) Cell extracts prepared from yeast strains expressing 3xFLAG-tagged α-subunits from their chromosomal loci were separated via blue native PAGE and immunoblotted with antibodies against FLAG. Samples were prepared under conditions favoring dissociation of the RP from CP from the 26S proteasome to directly examine subunit abundance in the context of the CP only. Triangles denote migrations of purified CP (filled) and a 20 kDa standard (open). Quantification of the percentage of free α-subunit (shown to the right) was determined by dividing the band corresponding to free α-subunit by the sum of the free and assembled (CP band) subunit abundance for each lane (*n* = 4; error bars = s.e.m.). ^, Blm10-CP; **, CP assembly intermediate. Full-length blot is presented in Supplementary Fig. S8. (**c**) Cell extracts prepared from yeast strains expressing the indicated 3xFLAG-tagged α-subunits from their chromosomal loci were fractionated by gel filtration chromatography. Peak fractions corresponding to free α-subunits and 26S proteasomes were separated via SDS-PAGE followed by immunoblotting with antibodies against FLAG. The percentage of free α-subunit (shown to the right) was determined by dividing the band corresponding to free subunit (free) by the sum of the free and 26S band intensities for each lane. (*n* = 8; error bars = s.e.m.). Full-length blot is presented in Supplementary Fig. S8.

We next examined the possibility that α1 may be quantitatively limiting for proteasome assembly. Although numerous groups have performed global measurements of proteasome subunit expression levels (discussed in Ho *et al*.^33^), a clear consensus on the relative abundance of α1 in yeast has not emerged. We hypothesized that if α1 were limiting for α-ring assembly, then it would be the least abundant α-subunit overall and would have the smallest amount of free, unincorporated subunit. To allow direct comparison of α-subunit abundance, we generated a panel of yeast strains in which each α-subunit was expressed as a 3xFLAG fusion from the endogenous chromosomal locus. These strains exhibited no obvious compensatory upregulation of other proteasome subunits (Fig. 7a, Rpt1 blot), and displayed no obvious growth defects under standard growth conditions, at elevated temperatures, or in the presence of known proteasome stressors *L*-canavanine or CdCl_2_ (Supplementary Fig. S6). This indicated that the 3xFLAG tags were well tolerated. We then separated whole cell extracts of each strain via SDS-PAGE and immunoblotted with anti-FLAG antibodies (Fig. 7a). Quantification of the resultant immunoblots revealed α1 to have the lowest total protein levels amongst the α-subunits, followed closely by α6. To assess the population of free subunit, we next separated extracts of the same strains via blue native PAGE and immunoblotted with anti-FLAG antibodies to visualize both free and incorporated α-subunits simultaneously (Fig. 7b). Quantification of the bands corresponding to the free subunits and 20S CP revealed that the majority of α1 is incorporated into the CP and is the least abundant free α-subunit, again closely followed by α6. As a complimentary approach, the same cell extracts were fractionated by gel filtration chromatography, and peak fractions corresponding to free subunit or 26S were separated via SDS-PAGE and immunoblotted with anti-FLAG antibodies (Fig. 7c). Similar to our results obtained via blue native PAGE, quantification of the free subunits and 26S proteasomes revealed that α1 was the least abundant free α-subunit. These data indicate that α1 is the stoichiometrically limiting α-subunit. Taking all of our results together, we propose that α1 protein levels limit proteasome abundance under normal growth conditions, and importantly, that elevated α1 levels favor formation of canonical CPs over non-canonical CPs when Pba3-4 chaperone activity is limiting.

## Discussion

To our knowledge, this work represents the first genome-wide analysis of genes influencing the subunit composition of the CP. Our use of a *pba3Δ* yeast strain allowed us to identify genetic determinants enhancing the ratio of canonical *versus* non-canonical CPs under conditions of limiting chaperone activity, similar to those occurring in response to some environmental stimuli. Our findings that elevated α1 preferentially promotes canonical CP assembly implies that the basal α1 expression level dictates how efficiently α4-α4 CPs form when chaperone activity is limiting. This is consistent with our findings that α1 is stoichiometrically limiting for CP assembly in yeast, and that overproduction of α1 enhances basal proteasome levels and resistance to stress. Importantly, this work shows that the subunit composition of a multisubunit complex can be influenced by how efficiently other subunits are incorporated at distal sites.

**Figure 8:**
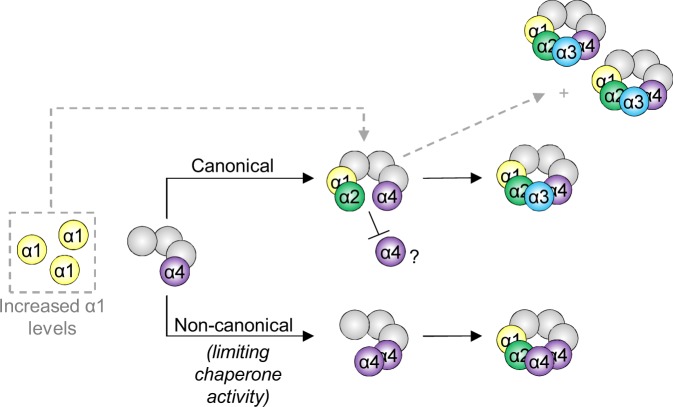
Hypothetical model for preferential assembly of canonical CPs upon α1 overexpression. Under limiting Pba3-4 chaperone activity (bottom pathway), non-canonical CPs can form in addition to canonical CPs (top pathway). Enhanced expression of α1 favors efficient incorporation of α1 and α2 prior to insertion of α3 or a second copy of α4 and confers preference for α3.

Assembly of the α-ring remains one of the most poorly understood aspects of proteasome biogenesis^16^. Recently, the order of α-subunit addition into the assembling α-ring was probed in human cells using systematic knockdown of each α-subunit with siRNA^31^. The authors demonstrated that a key step in α-ring assembly is the formation of a core assembly intermediate consisting of α4-α5-α6-α7. It was also shown that incorporation of α2 into the assembling α-ring was dependent upon the prior incorporation of α1. However, the order in which α1 and α3 were incorporated could not be determined. Our findings that enhancing α1 levels in *pba3Δ* cells preferentially promotes canonical CP formation suggests that α1 likely incorporates into assembling canonical α-rings prior to α3 (or a second α4), at least under our tested conditions. Further, it suggests that incorporation of α1 prior to a second copy of α4 favors, or possibly commits, the incorporation of α3 into the assembling α-ring over α4. Given that α4 poorly discriminates between α3 and α4 insertion at the α3 position during α-ring assembly^13^, we posit that α1 may preferentially promote canonical CP biogenesis by efficiently recruiting α2 to favor α3 incorporation (Fig. 8).

Overproduction of α1, but not other subunits, enhanced proteasome abundance and conferred resistance to common proteasome stresses in cells that assemble both canonical and non-canonical proteasomes (Figs 2 and 5). However, our data suggest that α1 is not rate-limiting for CP biogenesis (Fig. 6). Considering our findings that free α1 is the least abundant of the seven α-subunits, this suggests that incorporation of α1 is quantitatively limiting for CP biogenesis in yeast. Alternatively, α1 incorporation may also be thermodynamically unfavorable, and increasing α1 drives CP assembly by mass action. This is supported by the detection of some free subunit present in WT cells, and by our data demonstrating that α1 overproduction appears to enhance proteasome activity in *α3Δ* cells, as gauged by suppression of temperature and *L*-canavanine sensitivity. Similarly, whether α1 levels control basal proteasome levels in other species is not known.

There are multiple reports demonstrating the importance of subunit stoichiometry in proteasome assembly and cell health. In *Drosophila melanogaster*, the overexpression of the CP subunit β5 has been reported to significantly increase proteasome assembly and extend the lifespan of flies^34^. Similarly, increased expression of the RP subunit Rpn6 (PSMD11 in humans) has been demonstrated to enhance 26S proteasome formation and increase the longevity of human embryonic stem cells^35^. Both of these data strongly mirror our results in yeast upon α1 overexpression, where we observe a similar increase in proteasome assembly and enhanced growth under proteotoxic stress. Although neither β5 nor Rpn6 were identified as enhancers of proteasome assembly in our screen in *Saccharomyces cerevisiae*, these data, together with our findings, highlight a role for the stoichiometry of specific proteasome subunits in governing the efficiency of proteasome assembly.

An implication of our work is that the restricted expression of α1 is necessary to prime cells for alternative proteasome assembly. It is important to note that no physiological or environmental stimuli have yet been identified that exploit α1 stoichiometry to alter proteasome composition or stress resistance, either in yeast or in other organisms. We intend to investigate this in follow-up studies. However, we have made some interesting observations regarding the relationship between α1 and α4 levels in human cancers that support such a mechanism may exist. A previous analysis of publically available gene expression data revealed enhanced α4 expression and reduced α3 expression consistent with α4-α4 CP formation in two testicular cancer subtypes^14^. Prompted by this observation, we investigated the relationship between α1 expression and enhanced α4 expression in these same subtypes. We observed a significant decrease in α1 expression concomitant with enhanced α4 expression not only in these same subtypes, but in six additional datasets, comprising five testicular cancer subtypes (Supplementary Fig. S7). These data suggest that the stoichiometry of α1 may be altered in certain cancer types, specifically in testicular cancers, to enhance the assembly of non-canonical proteasomes in these cells. Considering that this was observed specifically in testicular cancers, it will also be interesting to see whether α1 levels influence the formation of α4s-containing spermatoproteasomes.

We found that the split-DHFR reporter system originally developed by Pelletier *et al*.^25^ can serve as a sensitive reporter of juxtaposition of subunits within a multisubunit complex. This basic approach could be readily adapted to search for genes regulating juxtaposition of other canonical or non-canonical subunit pairs. Several α-subunits undergo non-native interactions *in vitro* and *in vivo*^36–40^, and the cellular mechanisms that control or limit these pairings are unknown. Finally, it is noteworthy that other multisubunit complexes, such as the chaperonin complex TriC/CCT^41–43^ and the prefoldin chaperone complex^44^, undergo subunit substitutions to yield non-canonical complexes. This approach could be more broadly implemented to screen for genes, environmental stimuli, or small molecules that regulate alternative forms of these and other multisubunit complexes.

## Materials and Methods

### Yeast strains and media

Yeast manipulations were carried out according to standard protocols^45^. Strains used in this study are listed in Supplementary Table S1. For standard growth assays, the indicated strains were spotted onto the indicated media as six-fold serial dilutions prepared in water. For split-DHFR complementation growth assays, synthetic dropout plates lacking adenine were supplemented with 200 µM methotrexate hydrate (Tokyo Chemical Industry, Cat# 59-05-2) and 5 mg/mL sulfanilamide (Alfa Aesar, Cat# 63-74-1).

### Plasmids

All plasmids were constructed using standard molecular cloning techniques using TOP10F’ (Life Technologies) as a host strain. Plasmids used in this study are listed in Supplementary Table S2. Complete sequences and construction details are available upon request.

### Genomic tiling library screen

The Yeast Genomic Tiling Collection (Dharmacon, Cat# YSC4613) is comprised of 1,588 bacterial isolates each harboring a 2-micron plasmid containing a particular yeast genomic fragment, arrayed in 96-well plates^29^. To simplify the screening process, these isolates were grown on LB-kanamycin medium in a 96-well style array and subsequently scraped from the media and mixed prior to plasmid DNA isolation. This yielded 17 plasmid mixtures, which were then used to transform the *α2-α3 [DHFR] pba3Δ* reporter strain (RTY1304). Ninety percent of the cells were plated on media lacking leucine and containing 150 µM methotrexate and 5 mg/mL sulfanilamide to identify plasmids promoting DHFR complementation, and ten percent were plated on complete medium lacking leucine to estimate the fold library coverage. The genomic fragments contained within recovered plasmids were identified by DNA sequencing. Complete ORFs within the encoded genomic fragments were subcloned with 5′ and 3′ regulatory sequences into individual 2-micron plasmids and re-transformed into the *α2-α3 [DHFR] pba3Δ* reporter strain to identify the gene products.

### SDS-PAGE analysis

Cell extracts were prepared from equal numbers of cells via the alkaline lysis method^46^ and cleared via centrifugation at 21,000 × *g* for 30 seconds. Equal sample volumes were loaded onto 12% denaturing Tris-glycine SDS-PAGE gels and separated at 200 V at room temperature. After electrophoresis, proteins were transferred to PVDF membranes (EMD Millipore) at 100 V for one hour at 4 °C before immunoblotting with the indicated antibodies.

### Antibodies and immunoblotting

Immunoblotting was performed with antibodies against FLAG (Sigma Aldrich, Cat# F3165, 1:5,000), G6PD (Sigma Aldrich, Cat# A9521, 1:20,000), TetraHis (Qiagen, Cat# 34670, 1:2,000), 20S CP subunits (Enzo Life Sciences, Cat# PW9355, 1:2,000), Rpt5 (Enzo Life Sciences, Cat# PW8245, 1:10,000), C-Terminal DHFR (Sigma Aldrich, Cat# D0942, 1:1,000), Rpn12^47^ (1:10,000), Rpt1 (generated from hybridomas described in Geng *et al*.^48^, 1:5,000), and α4/Pre6 (Gift from Dieter Wolf^49^, 1:5,000). Blots were imaged on a Bio-Rad ChemiDoc MP using HRP-conjugated secondary antibodies (GE Healthcare) and ECL reagent.

### Native PAGE analysis

Cell extracts were prepared and separated by native PAGE essentially as described previously^27^. Mid- to late-log phase cells (OD_600_ = 1.5–2.0) grown in YPD or the appropriate synthetic dropout medium were harvested by centrifugation at 5,000 × *g* for five minutes at 4 °C, followed by washing with ice-cold distilled H_2_O. Cell pellets were frozen in liquid nitrogen and ground into powder using a mortar and pestle. The resulting cell powder was thawed in an equal volume of Extraction Buffer (50 mM Tris∙HCl, pH 7.5, 5 mM MgCl_2_, 10% glycerol) supplemented with 1 mM ATP, aprotinin, leupeptin, pepstatin A, and PMSF and incubated with frequent vortexing for 10 minutes on ice. Extracts were centrifuged at 21,000 × *g* for 10 minutes at 4 °C to remove cell debris. Equal amounts of protein (as determined by BCA assay) were loaded onto 4% non-denaturing gels and separated at 100 V, 4 °C. After electrophoresis, proteins were transferred to PVDF membranes at 100 V for one hour at 4 °C before immunoblotting with the indicated antibodies.

### Blue native PAGE analysis

Blue native PAGE was performed essentially as described previously^50^ with some modifications. Cell extracts were prepared with BN Extraction Buffer (50 mM Tris∙HCl, pH 7.5, 50 mM NaCl, 10% glycerol) supplemented with aprotinin, leupeptin, pepstatin A, and PMSF as described for native PAGE. Equal amounts of protein (determined by BCA assay) were loaded onto 4–16% gradient tricine gels lacking aminohexanoic acid and separated at 100 V, 4 °C using Cathode Buffer B (50 mM tricine, 7.5 mM imidazole, 0.02% Coomassie blue G-250; pH = 7.0) and Anode Buffer (25 mM imidazole; pH = 7.0). Once samples entered the stacking gel, the voltage was increased to 180 V. When the dye front migrated through ~1/3 of the resolving gel, Cathode Buffer B was exchanged for Cathode Buffer B/10 (50 mM tricine, 7.5 mM imidazole, 0.002% Coomassie blue G-250; pH = 7.0). After electrophoresis, proteins were transferred to PVDF membranes at 30 V overnight at 4 °C. The membranes were incubated in 100% methanol post-transfer to remove the majority of the Coomassie blue G-250 before immunoblotting with the indicated antibodies. The percentage of each free α-subunit was determined by dividing the band corresponding to the free subunit by the sum of the free subunit and band corresponding to the CP.

### Gel filtration chromatography

Whole-cell extracts were fractionated essentially as described previously^51^ with some modifications. Mid- to late log phase cells (OD_600_ = 1.5–2.0) grown in YPD were harvested by centrifugation at 5,000 × *g* for five minutes at 4 °C, followed by washing with ice-cold distilled H_2_O. Cell pellets were frozen in liquid nitrogen and ground into powder using a mortar and pestle. The resulting cell powder was thawed in an equal volume of Buffer A (50 mM Tris∙HCl, pH 7.5, 150 mM NaCl, 5 mM MgCl2, 10% glycerol, 1 mM ATP) and incubated with frequent vortexing for 10 minutes on ice. Extracts were centrifuged at 21,000 × *g* for 10 minutes at 4 °C to remove cell debris. Protein concentration was determined by BCA assay, and 2 mg of protein was fractionated at 4 °C on a Superose 6 10–30 column equilibrated in Buffer A supplemented with 1 mM ATP using an ÄKTA FPLC system (GE Healthcare). The resulting fractions were concentrated via acetone precipitation by addition of 6 μL of BSA (10 mg/ml) and 6 μL of 2% (w/v) sodium deoxycholate to 200 μL of each fraction. After incubation for 15 minutes at 4 °C, 1 mL of −20 °C acetone was added, mixed, and incubated at 4 °C for 2 hours. The precipitate was dried and dissolved in 40 μL of SDS sample buffer, and 10 μL were loaded onto 12% denaturing gels and separated at 200 V at room temperature. After electrophoresis, proteins were transferred to PVDF membranes at 100 V for one hour at 4 °C before immunoblotting with the indicated antibodies. The percentage of free α-subunit was determined by dividing the band corresponding to free subunit by the sum of the free subunit and subunit present in the 26S fraction for each lane.

### Disulfide crosslinking of α-subunits

Crosslinking of α-subunits was performed essentially as previously described^13–15,47,52^ with some modifications. Briefly, 20 OD_600_ equivalents of mid-log phase yeast cells expressing α-subunits with the desired cysteine substitutions were converted to spheroplasts with Zymolyase 20 T. Spheroplasts were then lysed in 0.15 mL of ice-cold Crosslinking Lysis Buffer (50 mM HEPES, pH 7.5, 150 mM NaCl, 5 mM MgCl_2_, 2 mM ATP). Lysis was achieved by vortexing three times at top speed for 30 second intervals with one minute incubations on ice in between. Cell debris was removed via centrifugation at 21,000 × *g* at 4 °C for 10 minutes, and 50 µL of supernatant was removed and added to 2.5 µL of 200 mM *N*-ethylmaleimide (final concentration of 10 mM) and 5.25 µL of 100 mM EDTA (final concentration of 10 mM). Disulfide crosslinking of 50 µL of the remaining extract was induced with 1.25 µL of 10 mM CuCl_2_ (final concentration of 250 µM) at 25 °C for 10 minutes, after which *N*-ethylmaleimide and EDTA were added as above. Samples were prepared with non-reducing sample buffer, loaded onto 10% denaturing Tris-glycine SDS-PAGE gels, and separated at 200 V at room temperature. After electrophoresis, proteins were transferred to PVDF membranes at 100 V for one hour at 4 °C before immunoblotting with the indicated antibodies. To reduce disulfide bonds, 1 µL of 1 M DTT (final concentration of 17 mM) was added to crosslinked samples for 10 minutes at room temperature prior to electrophoresis. The percentage of crosslinking was determined by dividing the band density corresponding to the crosslinked subunit by the sum of the densities of the crosslinked and uncrosslinked subunit for each lane.

### Kinetics of β5 propeptide cleavage

Yeast were grown to mid-log phase, at which time 14 OD_600_ equivalents were harvested by centrifugation at 4,122 × *g* for 5 minutes and resuspended in 7 mL of selective minimal medium. Cells were incubated at 30 °C for five minutes before adding 87.5 µL of 20 mg/mL CHX (final concentration of 250 μg/mL). Immediately after addition of CHX, 1.0 mL (2 OD_600_ equivalents) of culture was removed, added to 50 μL of ice-cold 200 mM sodium azide, and vortexed thoroughly to generate the zero time point sample. This procedure was repeated at the indicated time points following addition of CHX. At the conclusion of the chase, samples were centrifuged for 30 seconds at 10,000 × *g* to pellet the cells. Equal sample volumes were loaded onto 12% denaturing Tris-glycine SDS-PAGE gels and separated at 200 V. After electrophoresis, proteins were transferred to PVDF membranes at 100 V for one hour at 4 °C before immunoblotting with the indicated antibodies.

### Mass spectrometry

Yeast expressing β5-3xFLAG from the chromosomal locus in a *pba3Δ* background (RTY2263) were transformed with empty vector or a high-copy plasmid encoding α1. Cells were cultured (>30 cell doublings) in synthetic dropout medium supplemented with 30 mg/L light lysine (*L*-lysine monohydrochloride, Acros Organics, 657-27-2) or heavy lysine (*L*-Lysine-^13^C_6_, ^15^N_2_ dihydrochloride, Millipore Sigma, 608041) and harvested by centrifugation at 5,000 × *g* for five minutes at 4 °C, followed by washing with ice-cold distilled H_2_O. Cell pellets were frozen in liquid nitrogen and ground into powder using a mortar and pestle. The resulting cell powder was thawed in an equal volume of high-salt 20S Buffer (50 mM Tris∙HCl, pH 7.5, 5 mM MgCl_2_, 500 mM NaCl) and incubated with frequent vortexing for 10 minutes on ice. Extracts were centrifuged at 21,000 × *g* for 10 minutes at 4 °C to pellet insoluble material. The protein concentration of the supernatant was determined by BCA assay and equal protein amounts of light and heavy labeled supernatants were combined in a 1:1 ratio. The mixed sample was added to anti-FLAG M2 Affinity Gel (Sigma Aldrich, Cat# A2220) and incubated for 60 minutes at 4 °C. The resin was collected at 1,500 × *g* for two minutes at 4 °C, and the supernatant was decanted. The resin was then washed two times with high-salt 20S Buffer, followed by an additional wash in low-salt 20S Buffer (50 mM Tris∙HCl, pH 7.5, 5 mM MgCl_2_, 150 mM NaCl). Complexes were eluted from the resin with 200 μg/mL 3xFLAG peptide for 45 minutes at 4 °C. The eluted complexes were then concentrated in a 100,000 kDa MWCO filter (Sartorius, Cat# VS0141). The concentrated eluate was loaded onto a 4% non-denaturing gel and separated at 100 V, 4 °C, followed by gel staining with GelCode Blue (Thermo Fisher Scientific, Cat# 24590). Bands corresponding to 20S proteasomes were excised from gels and submitted to the FSU-COM Translational Science Laboratory for in-gel trypsinization and analysis by LC-MS/MS using a Q Exactive HF Hybrid Quadrupole-Orbitrap Mass Spectrometer (Thermo Fisher Scientific). Raw data was analyzed using MaxQuant and searched against the *Saccharomyces* Genome Database. The mean ratio of the CP subunits was calculated from the intensities of heavy *versus* light peptides. The intensity ratio of α4 was determined to be statistically different from the mean of the other CP subunits using an outlier test in GraphPad Prism.

### Microscopy

Cells were collected via centrifugation at 10,000 × *g* for 30 seconds, resuspended in YPD at 1/10th of the original culture volume, and applied to microscope slides overlaid with a thin pad of 3% agarose prepared in YPD to minimize cell movement during imaging. Slides were imaged using the EVOS FL Cell Imaging System (Thermo Fisher Scientific) equipped with a GFP filter set. Identical exposure times and light intensities were used for each sample.

### Quantification and statistical analysis

ImageLab (BioRad) was used for the quantification of band intensities. Statistical analysis was carried out using Graph Pad Prism 7.0 software via two-way ANOVA with Tukey’s test for multiple comparisons (Figs 3a and 5d), two-tailed t-test (Fig. 4a,c), or outlier test (Fig. 3b).

## Supplementary information

Supplementary Information for “Proteasome subunit α1 overexpression preferentially drives canonical proteasome biogenesis and enhances stress tolerance in yeast”

## Data Availability

All data generated or analyzed during this study are included in this published article (and its Supplementary Information files).
