# Supplementary material for: Proteasome subunit α1 overexpression preferentially drives canonical proteasome biogenesis and enhances stress tolerance in yeast
Source: Sci Rep. 2019 Aug 27;9:12418. doi: 10.1038/s41598-019-48889-5 (PMC6712033; doi:10.1038/s41598-019-48889-5)
Supplement: Supplementary file 1 — Supplementary Information for “Proteasome subunit α1 overexpression preferentially drives canonical proteasome biogenesis and enhances stress tolerance in yeast” [file 41598_2019_48889_MOESM1_ESM.pdf]

Supplementary Information

Proteasome subunit α1 overexpression preferentially drives canonical proteasome biogenesis and enhances stress tolerance in yeast

Lauren A. Howell, Anna K. Peterson, and Robert J. Tomko Jr.*

Department of Biomedical Sciences, Florida State University College of Medicine, Tallahassee, Florida 32306, USA

*Correspondence and requests for materials should be addressed to R. J. T., Jr. (email: robert.tomko@med.fsu.edu)

Howell *et al*, Supplementary Figure S1
Data related to Figure 1 in main text

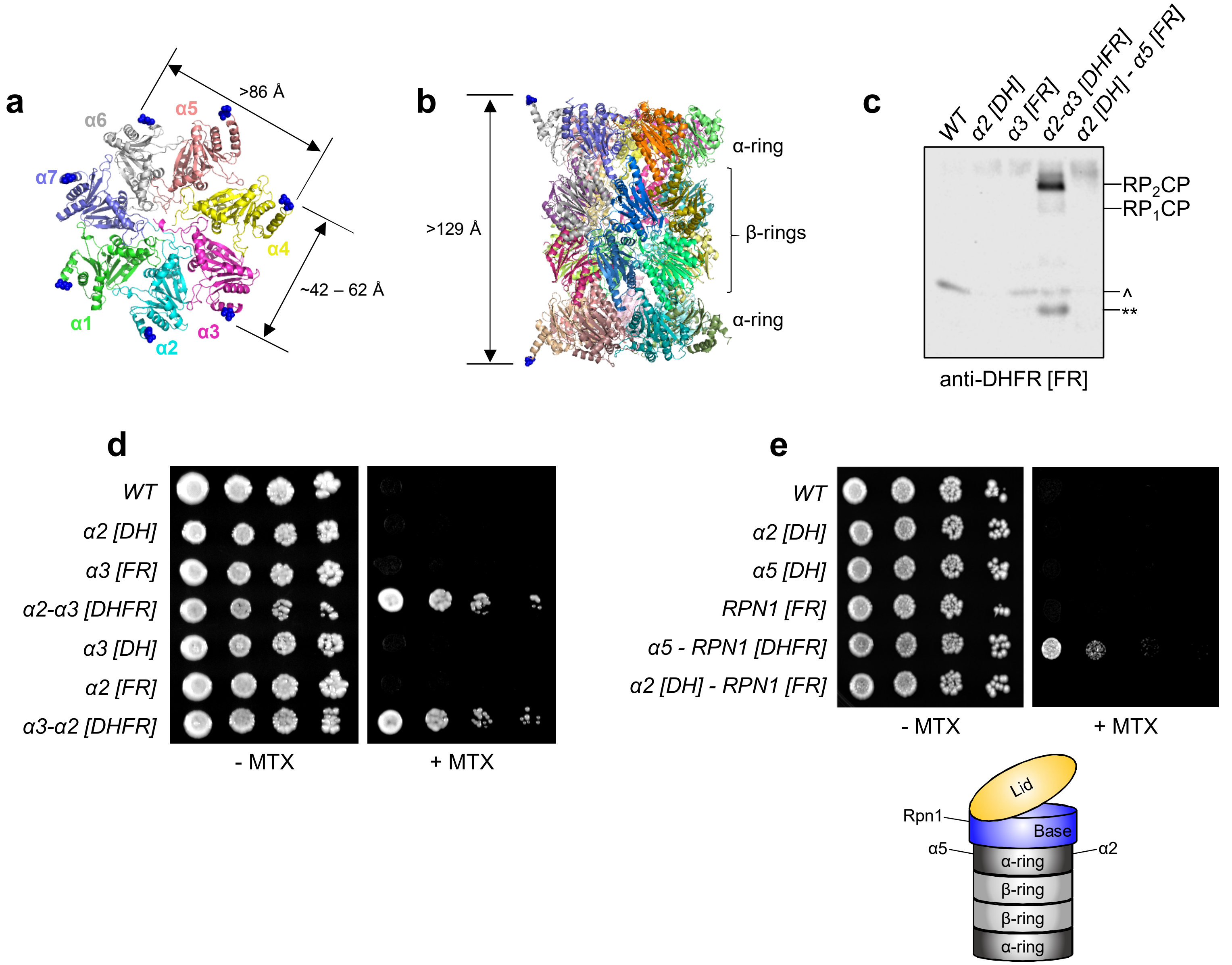

Howell *et al*, Supplementary Figure S2

Data related to Figure 2 in main text

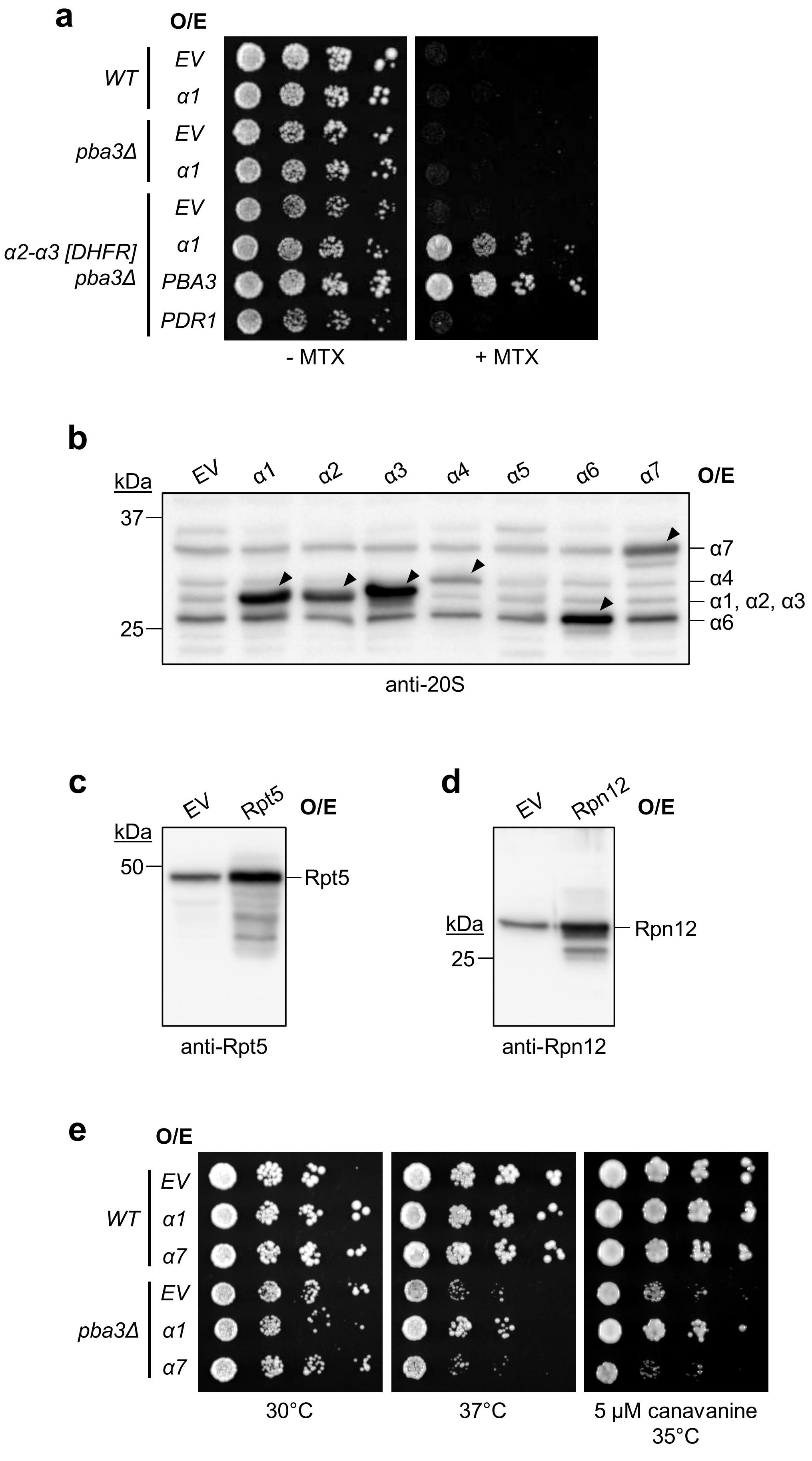

Howell *et al*, Supplementary Figure S3

Data related to Figure 3 in main text

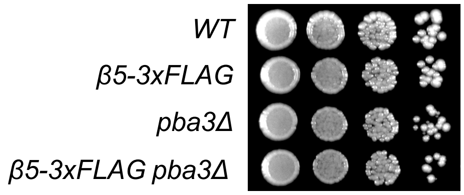

YPD, 30°C

Howell *et al*, Supplementary Figure S4
Data related to Figure 5 in main text

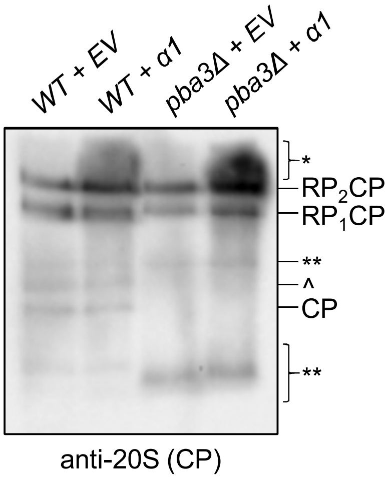

Howell *et al*, Supplementary Figure S5
Data related to Figure 6 in main text

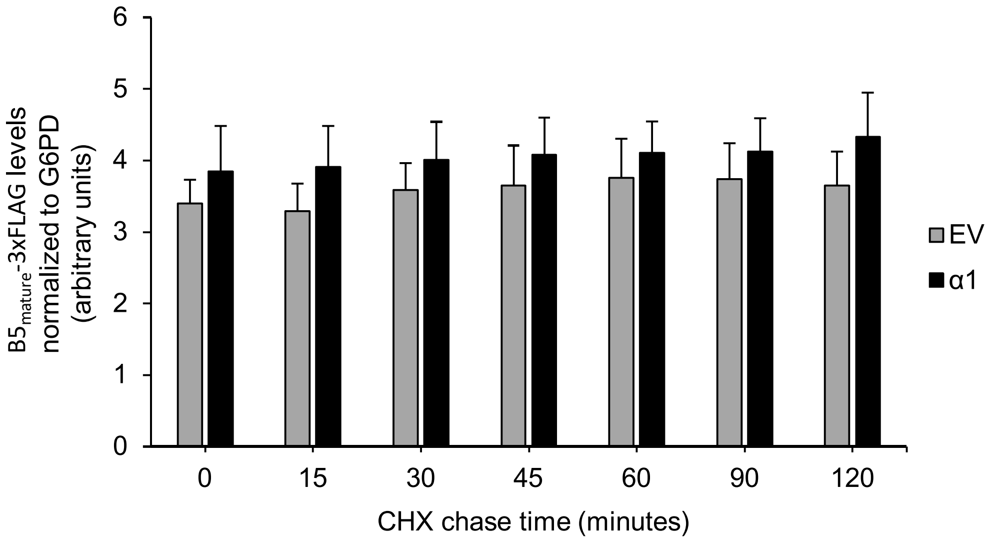

Howell *et al*, Supplementary Figure S6
Data related to Figure 7 in main text

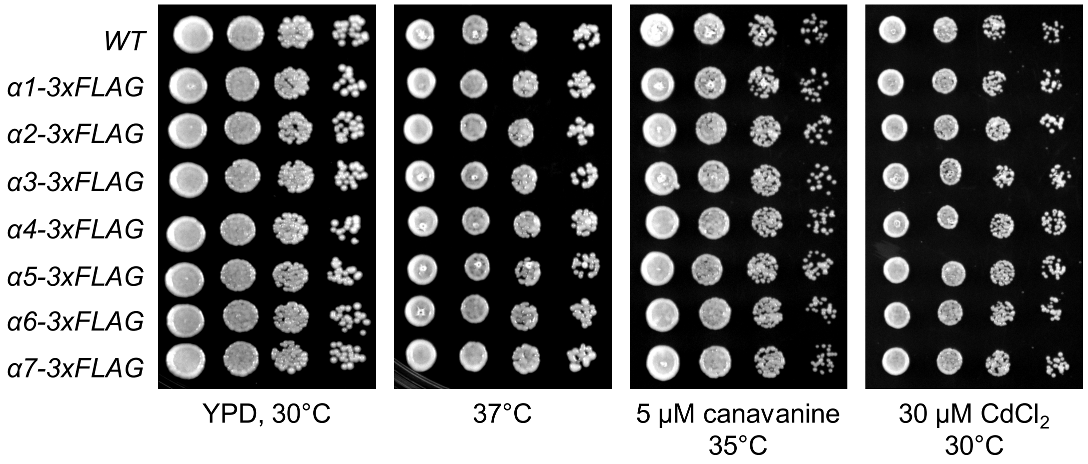

Howell *et al*, Supplementary Figure S7
Data related to Discussion in main text

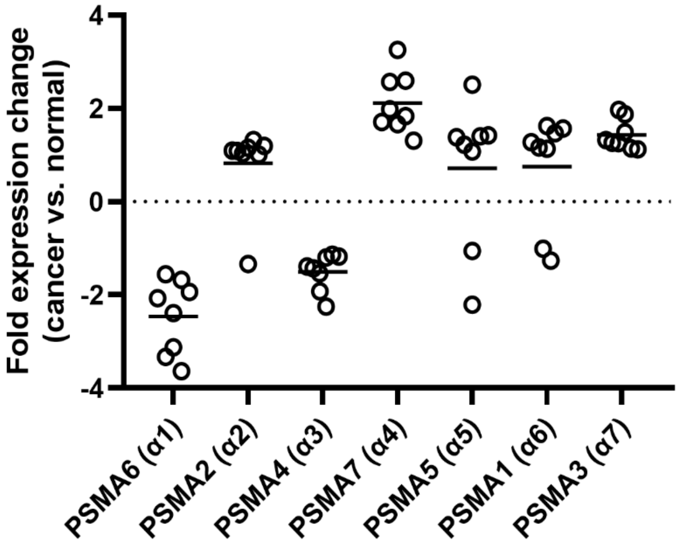

Howell *et al*, Supplementary Figure S8
Full-length blots/gels for main text figures

Fig. 1d - 20S

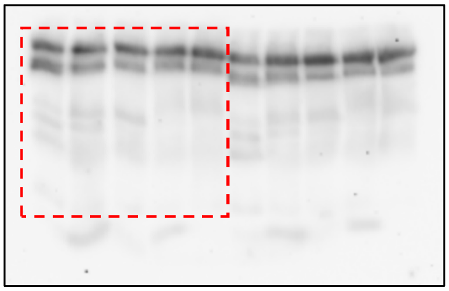

Fig. 1d - Rpn12

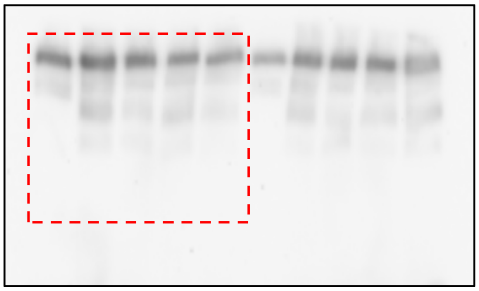

Fig. 3a

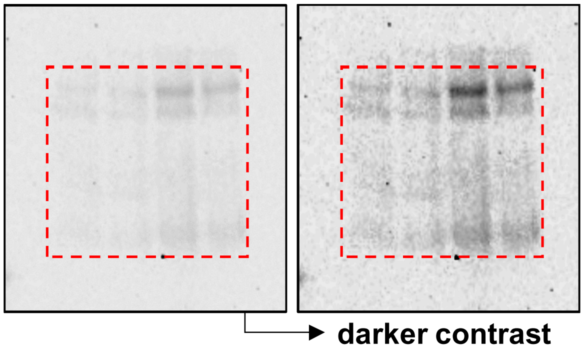

Fig. 3b

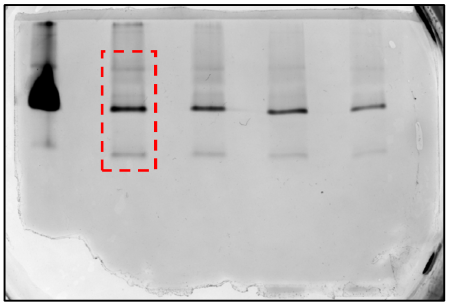

Fig. 4a

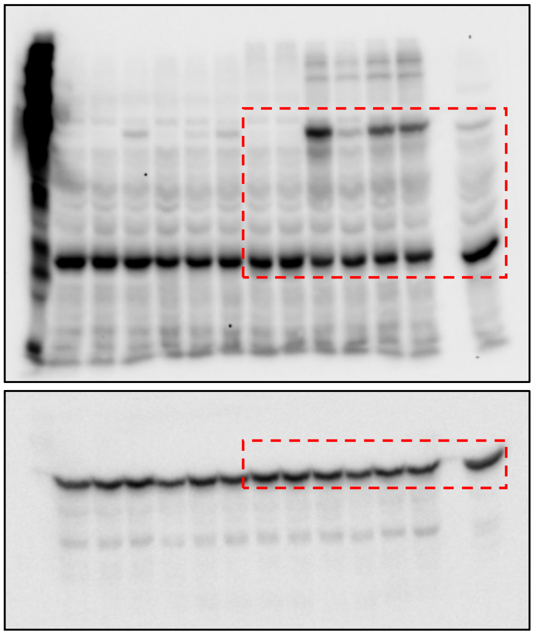

Fig. 4c

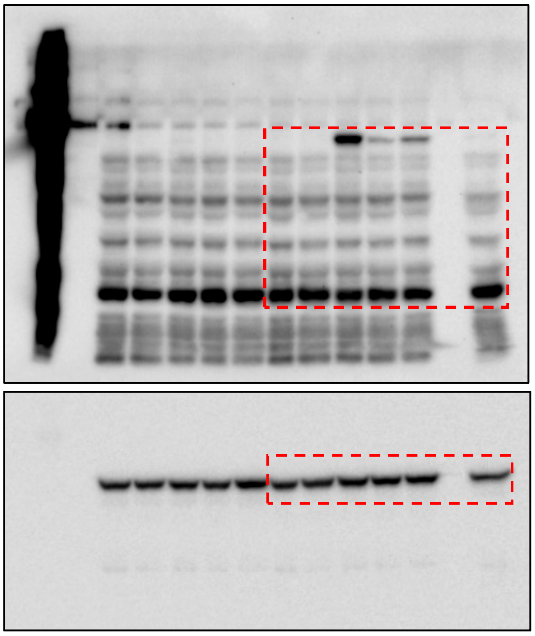

Fig. 5d

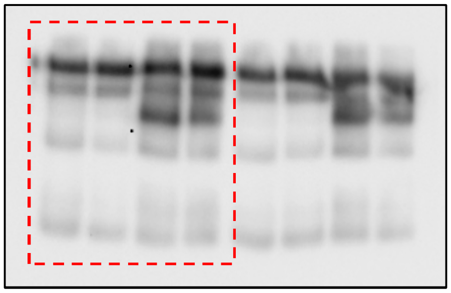

Fig. 6

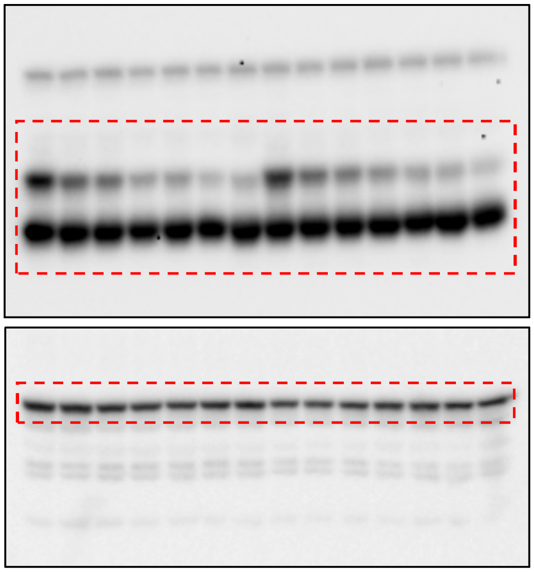

Fig. 7a

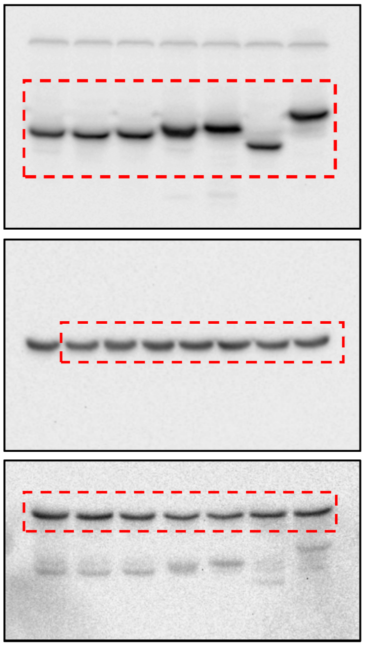

Fig. 7b

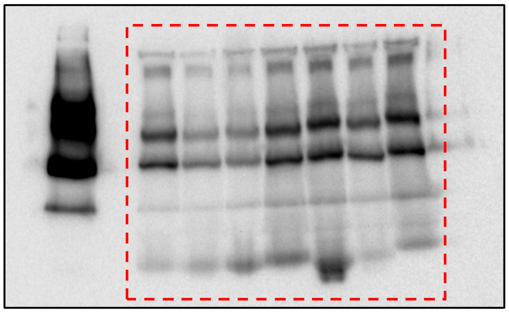

Fig. 7c

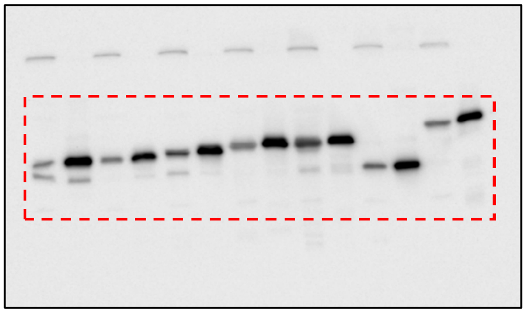

Howell *et al*, Supplementary Figure S9
Full-length blots for Supplementary Figures

Supplementary Fig. S1c

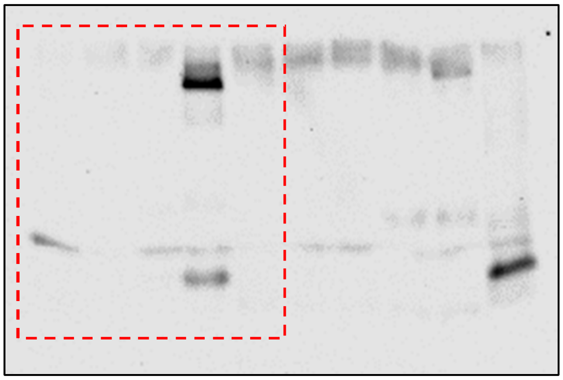

Supplementary Fig. S2b

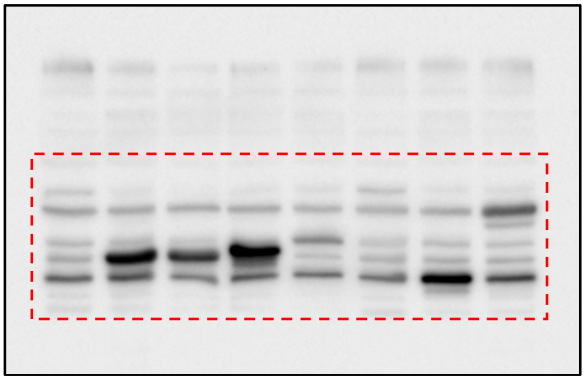

Supplementary Fig. S2c

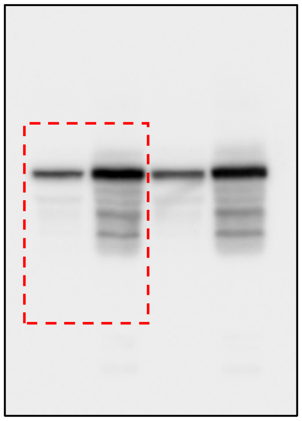

Supplementary Fig. S2d

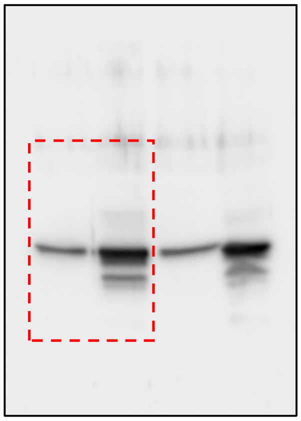

Supplementary Fig. S4

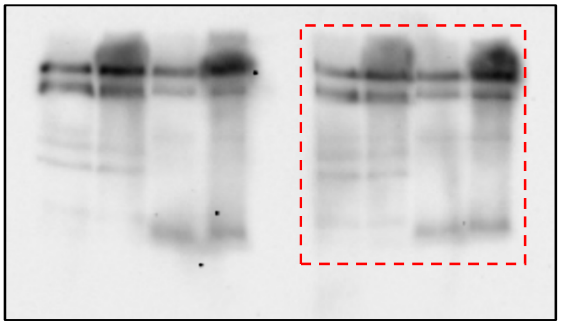

Supplementary Figure Legends

Supplementary Figure S1 related to Figure 1 in main text. (**a**) Estimation of intra-ring distance constraints for design of split-DHFR reporter system. A top view of the α-ring (PDB 1RYP) is shown, with the C-terminal amino acids of each subunit displayed as blue spheres. Example measurements of the distance between the C-terminal carboxylate carbons of nearest-adjacent neighbor and nearest-nonadjacent neighbor subunits are shown. Nearest-adjacent measurements spanned ~42-62 Å, whereas all nearest-nonadjacent measurements were greater than 86 Å. (**b**) Estimation of inter-ring distance constraints for design of split-DHFR reporter system. A side view of the α-ring (PDB 1RYP) is shown, with the C-terminal amino acids of opposing α-subunits displayed as blue spheres. An example measurement of the distance between the nearest inter-ring α-subunits is shown. All such measurements exceeded 129 Å. (**c**) Cell extracts from yeast strains expressing α-subunit fusions to N- or C-terminal DHFR fragments (designated [DH] or [FR], respectively) from their chromosomal loci were separated by non-denaturing PAGE and immunoblotted with antibodies against the C-terminal DHFR fragment. The positions of doubly-capped CP (RP_2CP) and singly-capped CP (RP_1CP) are shown. Under non-denaturing conditions, the [FR] fragment appears to be cleaved from subunits, but is stabilized upon complementation by an adjacent α-subunit [DH] fusion. **, possible CP assembly intermediate; ^, cross-reactive species. Full-length blot is presented in Supplementary Fig. S9. (**d,e**) Equal numbers of cells from the indicated yeast strains were spotted in six-fold serial dilutions on synthetic complete plates lacking or containing MTX and incubated for three days at 30°C. The illustration of the 26S proteasome accompanying (**e**) depicts the location of the RP base subunit Rpn1 relative to the α-subunits α2 and α5.

Supplementary Figure S2 related to Figure 2 in main text. (**a**) Overexpression of *PDR1* does not confer MTX resistance. The gene encoding *PDR1*, a transcription factor that regulates the pleiotropic drug response, was also encoded by the genomic fragment containing *α1*. To test whether the enhanced growth on MTX could be due to Pdr1-dependent efflux of MTX from cells, *the α2-α3 [DHFR] pba3Δ* reporter strain was transformed with a high-copy plasmid encoding *PDR1*. Transformants were spotted in six-fold serial dilutions onto synthetic complete plates lacking or containing MTX and incubated for three days at 30°C. Overexpression of Pdr1 did not confer growth on MTX. The growth assay shown here is an uncropped form of the image shown in **Fig. 2b in main text**. EV, empty vector. (**b-d**) Whole cell extracts were prepared from *pba3*Δ cells transformed with high-copy plasmids encoding the indicated proteins (**from Fig. 2c in main text**), followed by SDS-PAGE and immunoblotting with antibodies against the 20S CP (**b**), the RP base subunit Rpt5 (**c**), or the RP lid subunit Rpn12 (**d**). The 20S antibody does not recognize the α5 subunit. EV, empty vector. Full-length blots are presented in Supplementary Fig. S9. (**e**) Equal numbers of cells from the indicated yeast strains expressing empty vector (EV), α1, or α7 from a high-copy plasmid were spotted in six-fold serial dilutions on the indicated media and incubated as specified for three days.

Supplementary Figure S3 related to Figure 3 in main text. Equal numbers of cells from the indicated yeast strains were spotted in six-fold serial dilutions on YPD plates and incubated for two days at 30°C.

Supplementary Figure S4 related to Figure 5 in main text. Cell extracts of WT or *pba3Δ* yeast expressing empty vector (EV) or α1 from a high-copy plasmid were separated by non-denaturing PAGE and immunoblotted with antibodies against the 20S CP. The positions of doubly-capped CP (RP_2CP), singly-capped CP (RP_1CP), and CP are shown. **, CP assembly intermediates; ^, Blm10-CP; *, cross-reactive species that forms upon exogenous expression of α1. Full-length blot is presented in Supplementary Fig. S9.

Supplementary Figure S5 related to Figure 6 in main text. Quantification of mature β5 levels throughout the course of the cycloheximide (CHX) chase shown in **Fig. 6 in main text**. Bands corresponding to the mature species ($\beta5_{mature}$-3xFLAG) were normalized to the G6PD loading control (*n* = 8; error bars = s.e.m.).

Supplementary Figure S6 related to Figure 7 in main text. Equal numbers of cells from the indicated yeast strains were spotted in six-fold serial dilutions on the indicated media and incubated for two days as specified.

Supplementary Figure S7 related to Discussion in main text. Differential mRNA expression of proteasome α-subunits was examined in eight published testicular cancer datasets exhibiting elevated α4 mRNA levels (Korkola et al., 2006 (mixed germ cell tumor, embryonal carcinoma, seminoma, yolk sac tumor, and teratoma); Skotheim et al., 2005 (embryonal carcinoma, yolk sac tumor, and teratoma)) using Oncomine (www.oncomine.org). Data points represent the fold change in expression of a given gene for each cancer subtype, calculated via "cancer vs. normal" analysis (Rhodes et al., 2007). The means for each α-subunit is shown as a horizontal bar

Supplementary Figure S8: Full-length blots/gels for main text figures. Red boxes indicate the cropped portion of the blot/gel used in the figure.

Supplementary Figure S9: Full-length blots for Supplementary Figures. Red boxes indicate the cropped portion of the blot used in the figure.

Supplementary Table S1: Yeast strains used in this study

Name	Genotype	Source
RTY1	*MATa his3-Δ200 leu2-3,112 ura3-52 lys2-801 trp1-1* (alias MHY500)	Chen et al., 1993
RTY2	*MATα his3-Δ200 leu2-3,112 ura3-52 lys2-801 trp1-1* (alias MHY501)	Chen et al., 1993
RTY7	*MATa ura3-52 leu2-Δ1 his3-Δ200 trp1-Δ63 lys2-801 ade2-101* (alias YPH499)	Sikorski and Hieter, 1989
RTY25	*MATa ura3-52 leu2-Δ1 his3-Δ200 trp1-Δ63 lys2-801 ade2-101 ump1Δ::HIS3*	This study
RTY361	*MATα ura3-52 leu2-Δ1 his3-Δ200 trp1-Δ63 lys2-801 ade2-101 RPN1-GFP(S65T):kanMX6*	This study
RTY978	*MATα his3-Δ200 leu2-3,112 ura3-52 lys2-801 trp1-1 PRE6-yEGFP:kanMX6*	Nemec et al., 2017
RTY1008	*MATa his3-Δ200 leu2-3,112 ura3-52 lys2-801 trp1-1 pba3Δ::kanMX4* (alias MHY4386)	Kusmierczyk et al., 2008
RTY1010	*MATa his3-Δ200 leu2-3,112 ura3-52 lys2-801 trp1-1 pba4Δ::hphMX4* (alias MHY4298)	Kusmierczyk et al., 2008
RTY1064	*MATa his3-Δ200 leu2-3,112 ura3-52 lys2-801 trp1-1 pre9Δ::natMX4*	This study
RTY1244	*MATa his3-Δ200 leu2-3,112 ura3-52 lys2-801 trp1-1 PRE8-DHFR[1,2]:natMX4*	This study
RTY1249	*MATa his3-Δ200 leu2-3,112 ura3-52 lys2-801 trp1-1 PRE9-DHFR[1,2]:natMX4*	This study
RTY1255	*MATa his3-Δ200 leu2-3,112 ura3-52 lys2-801 trp1-1 PRE9-DHFR[3]:hphMX4*	This study
RTY1257	*MATa his3-Δ200 leu2-3,112 ura3-52 lys2-801 trp1-1 PRE8-DHFR[3]:hphMX4*	This study
RTY1263	*MATa his3-Δ200 leu2-3,112 ura3-52 lys2-801 trp1-1 PRE8-DHFR[1,2]:natMX4 PRE9-DHFR[3]:hphMX4*	This study

RTY1274	*MATα his3-Δ200 leu2-3,112 ura3-52 lys2-801 trp1-1 PRE9-DHFR[1,2]:natMX4 PRE8-DHFR[3]:hphMX4*	This study
RTY1304	*MATa his3-Δ200 leu2-3,112 ura3-52 lys2-801 trp1-1 PRE8-DHFR[1,2]:natMX4 PRE9-DHFR[3]:hphMX4 pba3Δ::kanMX4*	This study
RTY1310	*MATa his3-Δ200 leu2-3,112 ura3-52 lys2-801 trp1-1 PRE8-DHFR[1,2]:natMX4 PRE9-DHFR[3]:hphMX4 nas2Δ::HIS3*	This study
RTY1337	*MATα his3-Δ200 leu2-3,112 ura3-52 lys2-801 trp1-1 pre6Δ::HIS3 [pRS317-pre6-N79C-6His] pre9Δ::HIS3* (alias MHY2896)	Velichutina et al., 2004
RTY1338	*MATα his3-Δ200 leu2-3,112 ura3-52 lys2-801 trp1-1 pre6Δ::HIS3 [pRS317-pre6-I155C-6His] pre9Δ::HIS3* (alias MHY2897)	Velichutina et al., 2004
RTY1339	*MATα his3-Δ200 leu2-3,112 ura3-52 lys2-801 trp1-1 pre6Δ::HIS3 [pRS317-pre6-N79C,I155C-6His]* (alias MHY2900)	Velichutina et al., 2004
RTY1340	*MATα his3-Δ200 leu2-3,112 ura3-52 lys2-801 trp1-1 pre6Δ::HIS3 [pRS317-pre6-N79C,I155C-6His] pre9Δ::HIS3* (alias MHY2901)	Velichutina et al., 2004
RTY1347	*MATa his3-Δ200 leu2-3,112 ura3-52 lys2-801 trp1-1 PRE8-DHFR[1,2]:natMX4 PRE9-DHFR[3]:hphMX4 pba1Δ::kanMX4*	This study
RTY1434	*MATα his3-Δ200 leu2-3,112 ura3-52 lys2-801 trp1-1 pre6Δ::HIS3 [pRS316-PRE6] pre9Δ::HIS3*	This study
RTY1446	*MATa his3-Δ200 leu2-3,112 ura3-52 lys2-801 trp1-1 PUP2-DHFR[1,2]:natMX4*	This study
RTY1454	*MATa his3-Δ200 leu2-3,112 ura3-52 lys2-801 trp1-1 PUP2-DHFR[3]:hphMX4*	This study
RTY1479	*MATa his3-Δ200 leu2-3,112 ura3-52 lys2-801 trp1-1 PUP2-DHFR[1,2]:natMX4 PRE9-DHFR[3]:hphMX4*	This study
RTY1637	*MATα his3-Δ200 leu2-3,112 ura3-52 lys2-801 trp1-1 pre6Δ::HIS3 [pRS316-PRE6]*	This study
RTY1680	*MATa his3-Δ200 leu2-3,112 ura3-52 lys2-801 trp1-1 PRE9-DHFR[1,2]:natMX4 pre6Δ::HIS3 [pRS316-PRE6]*	This study
RTY1686	*MATa his3-Δ200 leu2-3,112 ura3-52 lys2-801 trp1-1 PUP2-DHFR[3]:hphMX4 pre6Δ::HIS3 [pRS316-PRE6]*	This study
RTY1693	*MATa his3-Δ200 leu2-3,112 ura3-52 lys2-801 trp1-1 SCL1-DHFR[1,2]:natMX4 PRE8-DHFR[3]:hphMX4*	This study

RTY1734	*MATa his3-Δ200 leu2-3,112 ura3-52 lys2-801 trp1-1 RPN1-DHFR[1,2]:natMX4*	This study
RTY1768	*MATa his3-Δ200 leu2-3,112 ura3-52 lys2-801 trp1-1 RPN1-DHFR[1,2]:natMX4 PUP2-DHFR[3]:hphMX4*	This study
RTY1884	*MATa his3-Δ200 leu2-3,112 ura3-52 lys2-801 trp1-1 PRE8-DHFR[1,2]:natMX4 RPN1-DHFR[3]:hphMX4*	This study
RTY2002	*MATα his3-Δ200 leu2-3,112 ura3-52 lys2-801 trp1-1 SCL1-DHFR[1,2]:natMX4 pre6Δ::HIS3 [pRS316-PRE6]*	This study
RTY2004	*MATα his3-Δ200 leu2-3,112 ura3-52 lys2-801 trp1-1 PRE8-DHFR[1,2]:natMX4 PUP2-DHFR[3]:hphMX4*	This study
RTY2116	*MATα his3-Δ200 leu2-3,112 ura3-52 lys2-801 trp1-1 PRE8-DHFR[1,2]:natMX4 PRE9-DHFR[3]:hphMX4 PRE6-yEGFP:kanMX6*	This study
RTY2117	*MATa his3-Δ200 leu2-3,112 ura3-52 lys2-801 trp1-1 PRE8-DHFR[1,2]:natMX4 PRE9-DHFR[3]:hphMX4 RPN1-GFP(S65T):kanMX6*	This study
RTY2205	*MATα his3-Δ200 leu2-3,112 ura3-52 lys2-801 trp1-1 pba3Δ::kanMX4 pre6Δ::HIS3 [pRS316-PRE6]*	This study
RTY2206	*MATa his3-Δ200 leu2-3,112 ura3-52 lys2-801 trp1-1 pre6Δ::HIS3 [pRS317-pre6-N79C,I155C-6His] pba3Δ::kanMX4*	This study
RTY2213	*MATa his3-Δ200 leu2-3,112 ura3-52 lys2-801 trp1-1 pre8-K160C-HF::URA3 pre9Δ::HIS3 [pRS315-pre9-L56C-T7] pba3Δ::kanMX4*	This study
RTY2214	*MATα his3-Δ200 leu2-3,112 ura3-52 lys2-801 trp1-1 pre8-K160C-HF::URA3 pre9Δ::HIS3 [pRS315-PRE9-T7]* (alias MHY1838)	Velichutina et al., 2004
RTY2215	*MATα his3-Δ200 leu2-3,112 ura3-52 lys2-801 trp1-1 PRE8-HF:URA3 pre9Δ::HIS3 [pRS315-pre9-L56C-T7]* (alias MHY1850)	Velichutina et al., 2004
RTY2216	*MATα his3-Δ200 leu2-3,112 ura3-52 lys2-801 trp1-1 pre8-K160C-HF::URA3 pre9Δ::HIS3 [pRS315-pre9-L56C-T7]* (alias MHY1839)	Velichutina et al., 2004
RTY2231	*MATa his3-Δ200 leu2-3,112 ura3-52 lys2-801 trp1-1 PRE2-6xGly-3xFLAG:HIS3MX6*	This study
RTY2263	*MATa his3-Δ200 leu2-3,112 ura3-52 lys2-801 trp1-1 PRE1-6xGly-3xFLAG:kanMX6 pba3Δ::kanMX4*	This study
RTY2320	*MATa his3-Δ200 leu2-3,112 ura3-52 lys2-801 trp1-1 SCL1-6xGly-3xFLAG:HIS3MX6*	This study

RTY2322	*MATa his3-Δ200 leu2-3,112 ura3-52 lys2-801 trp1-1 PRE8-6xGly-3xFLAG:HIS3MX6*	This study
RTY2324	*MATa his3-Δ200 leu2-3,112 ura3-52 lys2-801 trp1-1 PRE9-6xGly-3xFLAG:HIS3MX6*	This study
RTY2326	*MATa his3-Δ200 leu2-3,112 ura3-52 lys2-801 trp1-1 PRE6-6xGly-3xFLAG:HIS3MX6*	This study
RTY2328	*MATa his3-Δ200 leu2-3,112 ura3-52 lys2-801 trp1-1 PUP2-6xGly-3xFLAG:HIS3MX6*	This study
RTY2330	*MATa his3-Δ200 leu2-3,112 ura3-52 lys2-801 trp1-1 PRE5-6xGly-3xFLAG:HIS3MX6*	This study
RTY2332	*MATa his3-Δ200 leu2-3,112 ura3-52 lys2-801 trp1-1 PRE10-6xGly-3xFLAG:HIS3MX6*	This study
RTY2510	*MATa his3-Δ200 leu2-3,112 ura3-52 lys2-801 trp1-1 PRE6-6xGly-3xFLAG:HIS3MX6 pba3Δ::kanMX4*	This study

Supplementary Table S2: Plasmids used in this study

Name	Genotype	Source
pRT474	pRS424	Christianson et al., 1992
pRT711	pRS424-PRE8	This study
pRT845	pRS424-RPN12	This study
pRT1449*	pAG25-DHFR[1,2] (natMX4)	Tarassov et al., 2008
pRT1450*	pAG32-DHFR[3] (hphMX4)	Tarassov et al., 2008
pRT1513	pRS314-PRE6	This study
pRT1514	pRS315-PRE6	This study
pRT1515	pRS424-PRE9	This study
pRT1516	pRS424-PRE6	This study
pRT1517	pRS424-PRE10	This study
pRT1526	pRS314-PRE6-DHFR[1,2]	This study
pRT1527	pRS315-PRE6-DHFR[3]	This study
pRT1885	pRS424-RPT5	This study
pRT1964	pRS424-PBA3	This study

pRT1976	pRS424-SCL1	This study
pRT2202	pRS424-PDR1	This study
pRT2203	pRS424-DOA5	This study
pRT2204	pRS424-PRE5	This study

* The linker sequence for pRT1449 and pRT1450 is as follows: GGCGGTGGCGGATCAGGAG GCGGTGGGTCT, encoding amino acids GGGGSGGGGS.

Supplementary References

Chen, P., Johnson, P., Sommer, T., Jentsch, S. & Hochstrasser, M. Multiple ubiquitin-conjugating enzymes participate in the *in vivo* degradation of the yeast MAT alpha 2 repressor. *Cell* **74,** 357-369 (1993).

Christianson, T. W., Sikorski, R. S., Dante, M., Shero, J. H. & Hieter, P. Multifunctional yeast high-copy-number shuttle vectors. *Gene* **110,** 119-122 (1992).

Funakoshi, M., Tomko, R. J., Jr., Kobayashi, H. & Hochstrasser, M. Multiple assembly chaperones govern biogenesis of the proteasome regulatory particle base. *Cell* **137,** 887-899 (2009).

Korkola, J. E. et al. Down-regulation of stem cell genes, including those in a 200-kb gene cluster at 12p13.31, is associated with in vivo differentiation of human male germ cell tumors. *Cancer Research* **66,** 820-827 (2006).

Kusmierczyk, A. R., Kunjappu, M. J., Funakoshi, M. & Hochstrasser, M. A multimeric assembly factor controls the formation of alternative 20S proteasomes. *Nature Structural & Molecular Biology* **15**, 237 (2008).

Nemec, A. A., Howell, L. A., Peterson, A. K., Murray, M. A. & Tomko, R. J., Jr. Autophagic clearance of proteasomes in yeast requires the conserved sorting nexin Snx4. *The Journal of Biological Chemistry* **292,** 21466-21480 (2017).

Rhodes, D. R. et al. Oncomine 3.0: genes, pathways, and networks in a collection of 18,000 cancer gene expression profiles. *Neoplasia* **9,** 166-180 (2007).

Sikorski, R. S. & Hieter, P. A system of shuttle vectors and yeast host strains designed for efficient manipulation of DNA in Saccharomyces cerevisiae. *Genetics* **122,** 19-27 (1989).

Skotheim, R. I. et al. Differentiation of human embryonal carcinomas in vitro and in vivo reveals expression profiles relevant to normal development. *Cancer Research* **65,** 5588-5598 (2005).

Tarassov, K. et al. An *in vivo* map of the yeast protein interactome. *Science* **320,** 1465-1470 (2008).

Velichutina, I., Connerly, P. L., Arendt, C. S., Li, X. & Hochstrasser, M. Plasticity in eucaryotic 20S proteasome ring assembly revealed by a subunit deletion in yeast. *The EMBO Journal* **23,** 500-510 (2004).
